# An alternative pathway for delivery of power by outer hair cells to cochlear traveling waves

**DOI:** 10.1016/j.heares.2026.109618

**Published:** 2026-03-17

**Authors:** George Samaras, Julien Meaud

**Affiliations:** George W. Woodruff School of Mechanical Engineering, Georgia Institute of Technology, 771 Ferst Drive, Atlanta, 30332, GA, USA

**Keywords:** Cochlear mechanics, Cochlear amplifier, Outer hair cells, Micromechanics

## Abstract

*In vivo* measurements show that the basilar membrane (BM) exhibits sharp frequency tuning and high sensitivity to low-level stimuli. This has led most cochlear theories to assume that outer hair cells (OHCs) amplify traveling waves by delivering power directly to the BM. However, recent experiments revealed that the main bodies of Deiters cells (DCs), which are sandwiched between the OHCs and the BM, deform significantly in response to acoustic inputs. These findings challenge the hypothesis that power is transmitted by OHCs to the BM through the DCs. In this work, we consider a cochlear model that includes a micromechanical model of the organ of Corti with deformable DCs. The micromechanical model includes not only the BM, OHCs and DCs, but also other components of the organ of Corti, including the reticular lamina (RL) and pillar cells (PCs). We find that the amplitude and phase of the OHC-DC junction is consistent with *in vivo* measurements only if (1) the DC stiffness is comparable to that of OHCs; and (2) the joint between the RL and PCs is relatively stiff. Under these conditions, the model predicts that OHC electromotility does not deliver power to the BM through the classical OHC-DC-BM pathway, but rather through an alternative pathway through the RL and PCs. This result points to a new theoretical framework in which the RL and PCs, rather than DCs, serve as the primary conduit for the transfer of OHC-generated power to the BM and offers new insight into how the cochlea may achieve its remarkable sensitivity and frequency selectivity.

## 1. Introduction

Nonlinearity of the basilar membrane (BM) around the frequency of maximum response, called the best frequency (BF), is critical for normal hearing (Robles and Ruggero, 2001). This nonlinearity has been attributed to outer hair cells (OHCs), which have been hypothesized to supply power to cochlear traveling waves on a cycle-by-cycle basis (Ashmore et al., 2010; Dong and Olson, 2013). However, while the ability of OHCs to generate electromotile forces is well understood, how exactly these forces may amplify the low-level responses of the cochlea is currently under debate.

Classical theories of cochlear amplification assume that Deiters cells (DCs), which are sandwiched between the BM and OHCs, efficiently transmit OHC forces and power to the BM (Ramamoorthy et al., 2007; Wang et al., 2016; Sisto et al., 2019; Vencovsky` et al., 2019). However, the main bodies of DCs appear to deform significantly in response to acoustic inputs *in vivo* (Dewey et al., 2021; Strimbu et al., 2024) and to electrical inputs in a micro-chamber experiment on an isolated segment of the cochlear partition (Zhou et al., 2022). Furthermore, experimental estimates of the phase of DC elongation relative to the BM velocity are inconsistent with power delivery through DCs (Altoè et al., 2022). This raises the critical question of how OHC electromotile forces may deliver power to the BM.

Addressing this question requires one to examine the detailed anatomy and micromechanics of the organ of Corti (OoC) (Lim, 1986). The apical surfaces of OHCs form part of the reticular lamina (RL). The RL is directly connected to the pillar cells (PCs) which sit on top of the BM. The hair bundles (HBs) of the OHCs connect the tectorial membrane (TM) to the RL. It is commonly understood that the shear displacement of the OHC HBs stimulates mechano-electrical transduction channels, which gives rise to a voltage change in the main body of OHCs (Oghalai, 2004). This change of potential generates a cycle-by-cycle force due to electromotility. Recent measurements using optical coherence tomography (OCT) have revealed that OHC electromotility affects the vibrations of the different structural components of the OoC in a complicated manner *in vivo*. In particular, the nonlinearity of the response measured at the OHC-DC junction, TM (Chen et al., 2011; He et al., 2018; Dewey et al., 2021; Strimbu et al., 2024), and in some studies RL (He et al., 2018; Dewey et al., 2019) has a broader frequency range that extends to lower frequency than the nonlinearity observed at the BM. Furthermore, the motion at the OHC-DC junction lags the BM motion (Dewey et al., 2021). The motion at the upper part of the OoC (RL and TM) is also not in phase with the BM motion and has been found in multiple studies to lead the BM motion sub-BF (He et al., 2018, 2022; Dewey et al., 2021; Cho and Puria, 2022). Large tonic (direct current) displacements are observed at the RL, TM and OHC-DC junction, although the tonic displacements are negligible at the BM (Dewey et al., 2021). These recent experimental findings establish extensive constraints on OoC micromechanics that can be used to evaluate cochlear models.

**Fig. 1.**
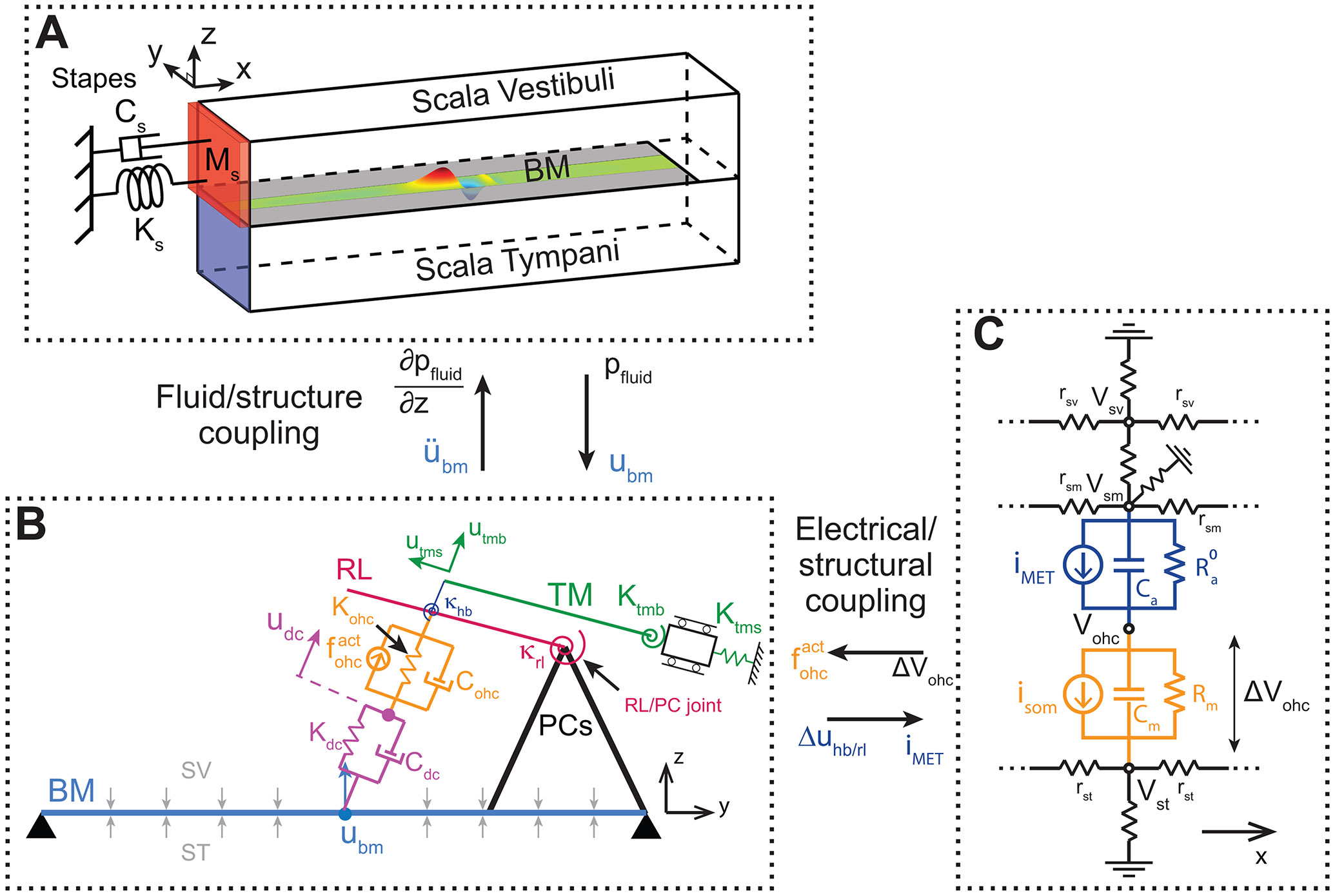
Schematic of the multiphysics cochlear model used in this study. A. Acoustical domain consisting of fluid in the scala vestibuli and scala tympani. B. Micromechanical model of the OoC. Elements within the OoC are modeled as lumped elements. The DCs are modeled as deformable elements with stiffness $K_{dc}$ and viscosity $C_{dc}$. The joint between the RL and PCs is modeled as a torsional spring of stiffness $\kappa_{rl}$. C. Electrical domain consisting of circuit representation of the OHCs and the fluids in the cochlear ducts. Quantities that couple the micromechanical domain to the acoustical and electrical domains, respectively, are shown between each panel.

The current article examines how OHCs may provide power to traveling waves if the recent findings regarding the compliance of the main body of DCs are taken into account. A previously developed computational model of the gerbil cochlea is used as the basis for this work (Samaras et al., 2023). In the previous model, the DCs were modeled as rigid links between the OHCs and the BM. This assumption is relaxed in the current model, which considers the DCs to be deformable viscoelastic elements (Fig. 1B). The model allows forces to be transmitted to the BM either through the DC or through the PCs. The influence of the stiffness of the DC and of the joint between the RL and PCs on the micromechanical response of the organ of Corti is examined. Numerical results are compared to available OCT measurements of OoC micromechanics to establish the set of parameters that result in predictions most similar to experimental results, before analyzing power transfer to the BM.

## 2. Methods

### 2.1. Model overview

This research is based on a previously developed model of the mammalian cochlea which has been presented in a series of papers (Ramamoorthy et al., 2007; Meaud and Grosh, 2010; Bowling and Meaud, 2018; Samaras et al., 2023). The model couples an acoustical model of the intracochlear fluid to a micromechanical model of the OoC (Fig. 1A-B). The fluid in the scala tympani and scala vestibuli, which is modeled as inviscid and incompressible, is coupled only to the BM. The lumped element representation of OoC micromechanics, which is described below, is coupled via electromotility and mechano-electrical transduction (MET) to an electrical model that represents the electrical potentials in the cochlear ducts and in the OHCs (Fig. 1C).

### 2.2. Kinematics of the organ of Corti

The previous version of the OoC model assumed the DCs to be rigid elements and had three structural degrees of freedom (DOFs) at each cross-section of longitudinal coordinate x: the BM transverse displacement at the BM midpoint, $U_{bm}(x)$, the TM bending displacement, $u_{tmb}(x)$, and the TM shearing displacement, $u_{tms}(x)$. In order to model the DCs as deformable elements, the new version of model includes an additional DOF at each cross-section: the axial displacement of the OHC-DC junction, $u_{dc}(x)$ (Fig. 1B). Readers interested in how this significant change in the model formulation was introduced should refer to the Supporting Material. Furthermore, while the previous model (Samaras et al., 2023) explicitly modeled the three rows of OHCs, the current model lumps the three rows of OHCs into a single row - a modification which we found to have negligible effects on model predictions.

The BM displacement is assumed to depend on the radial coordinate y according to:

(1) $${\vec{u}}_{bm}(x,y)=U_{bm}(x)\psi_{bm}(y)\vec{z}\;\text{for}-b(x)∕2\leq y\leq b(x)∕2,$$

where b(x) is the radial width of the BM and $\psi_{bm}(y)=\sin\left[\frac{\pi (y+b(x)∕2)}{b(x)}\right]$ is the mode shape for the BM displacement. The TM displacement at the tip of the OHC HB is given by:

(2) $${\vec{u}}_{tm}(x)=-u_{tms}(x){\vec{y}}_{rl}+u_{tmb}(x){\vec{z}}_{rl},$$

where ${\vec{y}}_{rl}$ is the unit vector in the RL direction and ${\vec{z}}_{rl}$ is the unit vector normal to the RL.

The vibrations of the OoC are assumed in the radial/transverse (yz) plane only, (*i.e.* there is no longitudinal component of vibration). Furthermore, the phalangeal processes of DCs are neglected, and the longitudinal tilt of OHCs ignored. Longitudinal coupling is considered in the mechanics of the BM and TM, as described in our previous work (Meaud and Grosh, 2010; Samaras et al., 2023). The model of the organ of Corti makes three important assumptions: the PCs, the RL, and the OHC HBs are rigid bodies. Details about the anatomical and kinematics models can be found in Section 1 of the Supplemental Material.

In addition to the BM and the TM, the OoC model includes four deformable elements at each cross-section: the axial springs that represent the axial stiffness of OHCs and DCs (of stiffness $K_{ohc}$ and $K_{dc}$, respectively), and the torsional springs of the RL to PC joints and of the HB to RL joint (of torsional stiffness $\kappa_{rl}$ and $\kappa_{hb}$, respectively). The potential energy densities of these elements are:

(3) $$v_{ohc}=\frac{N_{ohc}}{2}K_{ohc}{\left[{\Delta u}_{ohc}^{comp}\right]}^{2},$$

(4) $$v_{dc}=\frac{N_{ohc}}{2}K_{dc}{\left[{\Delta u}_{dc}^{comp}\right]}^{2},$$

(5) $$v_{rl}=\frac{1}{2}\kappa_{rl}{\left[\Delta \theta_{pc∕rl}\right]}^{2}=\frac{1}{2}K_{rl}{\left[\Delta u_{pc∕rl}\right]}^{2},$$

(6) $$v_{hb}=\frac{N_{ohc}}{2}\kappa_{hb}{\left[\Delta \theta_{hb∕rl}\right]}^{2}=\frac{N_{ohc}}{2}K_{hb}{\left[\Delta u_{hb∕rl}\right]}^{2},$$

where ${\Delta u}_{ohc}^{comp}$ is the compression of the OHC; ${\Delta u}_{dc}^{comp}$ is the DC compression; $\Delta \theta_{rl∕pc}$ is the RL rotation of the RL relative to the PC; and $\Delta \theta_{hb∕rl}$ is the HB rotation relative to the RL. $N_{ohc}=3$ is the number of OHCs (and DCs) per cross-section. It is convenient to replace the torsional springs $\kappa_{rl}$ and $\kappa_{hb}$ by equivalent linear springs of stiffness $K_{rl}=\kappa_{rl}∕L_{r0}^{2}$ and $K_{hb}=\kappa_{hb}∕L_{hb}^{2}$ associated with the deformations $\Delta_{pc∕rl}=L_{ipc}\Delta \theta_{pc∕rl}$ and $\Delta \theta_{hb∕rl}=L_{hb}\Delta \theta_{hb∕rl}$, where $L_{ipc}$ and $L_{hb}$ are the lengths of the inner PC and OHC HB, respectively.

As shown in Supplemental Material, the assumptions regarding OoC kinematics allow us to express the deformation of the springs as linear combinations of the displacement of the four DOFs:

(7) $$u_{def}=G^{e}u_{dof},$$

where $u_{def}={\left[\Delta u_{hb∕rl},\Delta u_{pc∕rl},{\Delta u}_{ohc}^{comp},{\Delta u}_{dc}^{comp}\right]}^{T}$, $u_{dof}={\left[U_{bm},u_{tms},u_{tmb},u_{dc}\right]}^{T}$, and $G^{e}$ is a 4 × 4 matrix that corresponds to the kinematic coupling between these elastic elements and the DOFs of the model. This expression enables us to derive the equations of motion of the system according to Lagrange’s equations, as described in Supplemental Material.

### 2.3. Viscous damping within the organ of Corti

The model includes viscous damping directly applied to the BM and two TM DOFs as well as viscous damping within the OoC. The model lumps the effect of damping due to fluid viscosity within the subtectorial space (STS) into the OoC model, by including a damping force proportional to the shear velocity of the TM relative to the RL ($\Delta {\dot{u}}_{sts}$). Furthermore, damping forces proportional to ${\Delta \dot{u}}_{ohc}^{comp}$ and ${\Delta \dot{u}}_{dc}^{comp}$ account for the viscosity of OHCs and DC, respectively. In a manner similar to Eq. (7), the relation between the time derivative of the deformation of viscous elements within the OoC and the velocity of the DOFs can be expressed as:

(8) $$v_{def}=G^{v}{\dot{u}}_{dof}$$

where $v_{def}={\left[\Delta {\dot{u}}_{sts},{\Delta \dot{u}}_{ohc}^{comp},{\Delta \dot{u}}_{dc}^{comp}\right]}^{T}$, and $G^{v}$ is a 3 × 4 matrix that corresponds to the kinematic coupling between the viscous elements within the OoC and the DOFs of the model.

### 2.4. Electromotility and mechano-electrical transduction

The electromotile force applied by OHCs is given by:

(9) $$f_{ohc}^{act}=\epsilon_{e}\cdot \Delta \phi_{ohc}$$

where $\epsilon_{3}$ is the electromotile coupling coefficient and $\Delta \phi_{ohc}$ is the perturbation in the transmembrane potential. The mechanoelectrical transduction (MET) current, $i_{MET}$, regulates the voltage across the OHCs and is a nonlinear function of the deflection of the hair bundle deflection relative to the RL, $\Delta u_{hb∕rl}$:

(10) $$i_{MET}(\Delta u_{hb∕rl})=G_{hb}^{max}\cdot \Delta V_{bh}^{0}\cdot \left[\frac{1}{1+\text{exp}(-\frac{\Delta u_{hb∕rl}-X_{0}}{\Delta X})}-P_{0}^{s}\right]$$

where $G_{hb}^{max}$ is the saturating conductance of the MET channels; $\Delta V_{hb}^{0}$ is the resting value of the difference between the scala media potential and the intracellular OHC potential; ΔX is a constant value; $P_{0}^{s}$ is the resting open probability of the HB MET channel; and $X_{0}=\text{ln}(1∕P_{0}^{s}-1)\cdot \Delta X$. Note that $G_{hb}^{max}$ depends on the longitudinal coordinate x. The baseline values of the MET conductance function, $G_{hb}^{max}(x)$, are listed in the Supplemental Material. As described in the Results section, the value of $G_{hb}^{max}(x)$ was scaled from its baseline value, $G_{hb,BL}^{max}(x)$, by multiplying $G_{hb,BL}^{max}(x)$ at basal locations (x ≤ 0.44 cm) by a constant scaling factor in some simulations in order to adjust the predicted BM gain.

### 2.5. Analysis of power transfer to the BM

**Fig. 2.**
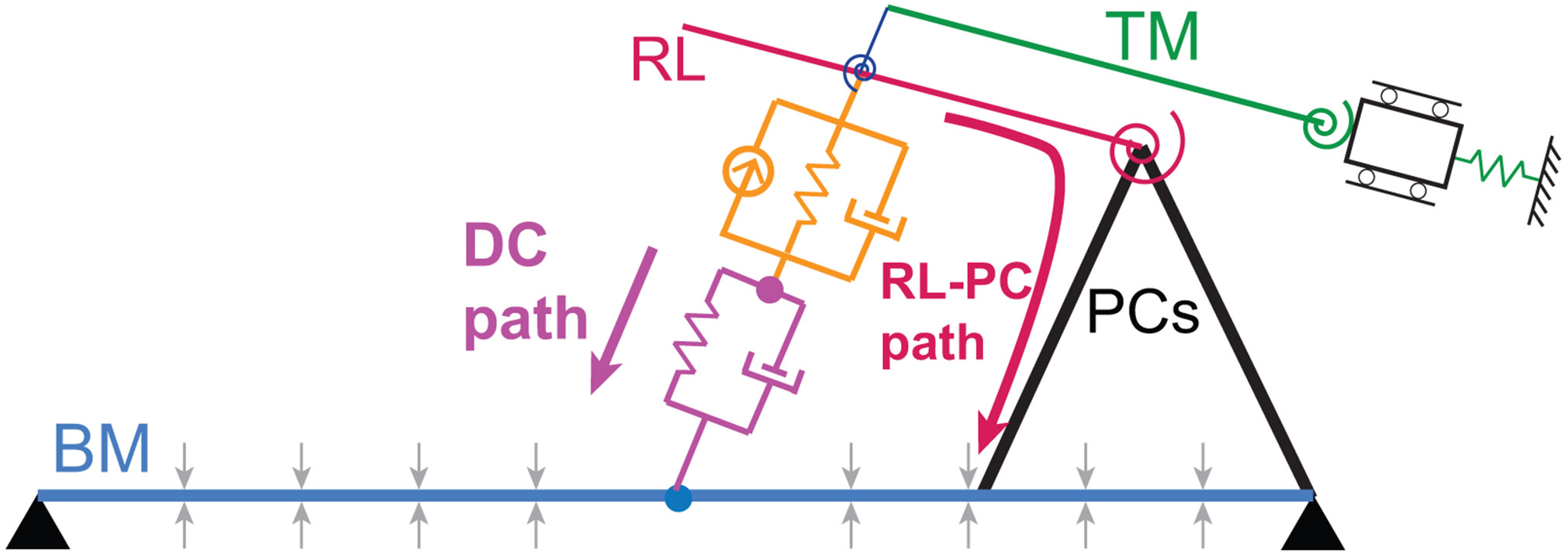
Diagram of organ of Corti with DC and RL/PC pathways for power delivery to the BM. DC pathway and RL-PC pathway are shown with thick magenta and dark red arrows, respectively.

In order to analyze power delivery to the BM, we need to examine all the forces applied by the OoC and by the fluid on the BM. Within the OoC, only the DCs and the PCs are directly connected to the BM and directly apply forces on the BM. Details about the calculation of the forces applied by DCs and PCs on the BM ($F_{dc∕bm}$ and $F_{pc∕bm}$, respectively) are given in the Supplemental Material. The DC force corresponds to what we call the DC pathway which is the classical way in which forces and power are delivered by DCs to the BM in most cochlear models (see the magenta arrow in Fig. 2). The force applied by the PCs on the BM forms what we call the RL-PC pathway (see the dark red arrow in Fig. 2) because these forces are transmitted to the BM due to the rigid coupling between the PCs and the BM.

Analysis of power delivery also requires us to consider the force applied by neighboring sections due to BM longitudinal coupling, $F_{bm}^{lc}$ (*i.e.* the terms that arise from ${ℒ}_{bm,2.5D}\left[U_{bm}(x)\right]$ in Eq. S30) and the force due to viscous damping on the BM, $F_{bm}^{v}$ (that is, proportional to the viscous damping coefficient of the BM, $C_{bm}$). Furthermore, the force applied by the fluid needs to be considered. The model includes a three-dimensional model of the fluid pressure in the scala tympani (ST) and scala vestibuli (SV). The modal force (per unit length) applied by the fluid on the BM mode is obtained by projecting the fluid pressure difference between the ST and SV onto the assumed mode shape for the BM:

(11) $$F_{f∕bm}(x)=\int_{-b(x)∕2}^{b(x)∕2}\left[p_{st}(x,y,z=0)-p_{sv}(x,y,z=0)\right]\psi_{bm}(x,y)dy.$$

These generalized forces from the OoC and fluid can be used to evaluate the power delivered to the BM. For time-harmonic responses with an $e^{j\omega t}$ time dependence, the power delivered to the BM by a force applied on the BM ($F_{j∕bm}$) is given by:

(12) $$\Pi_{j∕bm}=\frac{1}{2}R\left[F_{j∕bm}{\left(j\omega U_{bm}\right)}^{*}\right],$$

where ω is the angular frequency, R denotes the real part and ∗ the complex conjugate. This equation is equivalent to

(13) $$\Pi_{j∕bm}=\frac{1}{2}|F_{j∕bm}\;∥\;V_{bm}|\;\cos(∠F_{j∕bm}-∠V_{bm}),$$

where ∠ denotes the phase angle. This equation highlights the link between power delivery and the value of the phase difference between the force and velocity: power delivery ($\Pi_{j∕bm}>0$) requires that the phase difference $∠F_{j∕bm}-∠V_{bm}$ is between −0.25 and 0.25 cycles. Note that the total power delivered to the BM is equal to 0:

(14) $$\Pi_{dc∕bm}+\Pi_{pc∕bm}+\Pi_{bm}^{v}+\Pi_{bm}^{lc}+\Pi_{f∕bm}=0,$$

where $\Pi_{dc∕bm}$ and $\Pi_{pc∕bm}$ are the powers delivered by the DC pathway and the RL-PC pathway to the BM, respectively ; $\Pi_{bm}^{v}$ is the power due to viscous drag on the BM; $\Pi_{bm}^{lc}$ is the power delivered to the BM by neighboring sections due to BM longitudinal coupling; and $\Pi_{f∕bm}$ is the power delivered by the fluid on the BM. Eq. (14) implies that the BM delivers power to the fluid (or that the fluid absorbs power) $\Pi_{dc∕bm}+\Pi_{rl∕bm}$ is greater than the sum of the power dissipated by viscous drag on the BM and lost to neighboring cross-sections due to the longitudinal coupling. A negative value of the BM resistance is often used to evaluate power delivery to traveling wave. $\Pi_{f∕bm}$ is also related to the BM resistance according to the equation:

(15) $$\Pi_{f∕bm}=\frac{1}{2}R_{bm}\omega^{2}{|U_{bm}|}^{2},$$

where $R_{bm}$ is the BM resistance, which can be calculated as:

(16) $$R_{bm}=R\left[-\frac{F_{f∕bm}}{j\omega U_{bm}}\right].$$

### 2.6. Linear stability analysis

The model is formulated using a state-space approach, which allows us to simulate the nonlinear dynamics of the system in the time domain and to easily evaluate the linear stability (Elliott et al., 2007; Meaud and Lemons, 2015). The eigenvalues of the state-space matrix can be written as

(17) λ=σ±iω.

The sign of the real part σ indicates the stability of the corresponding eigenmode: a positive σ value corresponds to an unstable mode, *i.e.* an exponentially diverging response of frequency ω. While the linear frequency response of a model can be computed even if the model has a linear instability, this linear frequency response is only meaningful in the case of a linearly stable model as the response of an unstable model computed in the time domain would be exponentially divergent.

## 3. Results

### 3.1. Influence of the stiffness of DCs and RL/PC joint on BM response

**Fig. 3.**
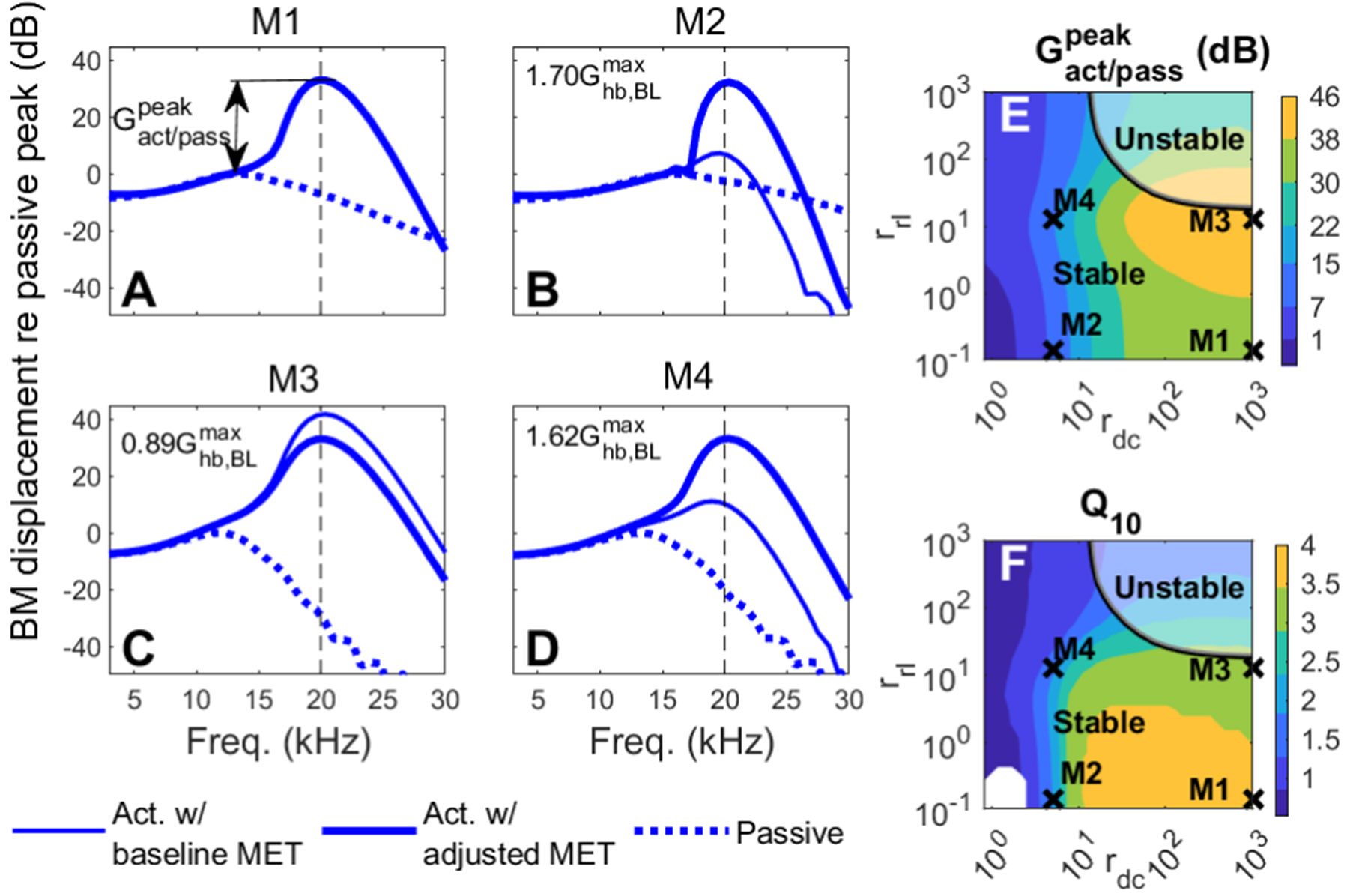
Influence of DC and RL stiffness on the linear response to a pure tone at the 20 kHz location. A-D show the BM displacement response to a pure tone for four models with different values for the stiffness of the DC and RL/PC joint: M1 ($r_{dc}$ = 1000 and $r_{rl}$ = 0.14); M2 ($r_{dc}$ = 5 and $r_{pc}$ = 0.14); M3 ($r_{dc}$ = 1000 and $r_{rl}$ = 12.33); M4 ($r_{dc}$ = 5 and $r_{lr}$ = 12.33). In A-D, the dotted line and the thin solid line are the responses of the passive model and of the active model with baseline MET conductance, respectively. In panels B-D, the thick solid line is the response of the active model with a saturating MET conductance adjusted so that the model has the same active to passive gain as model M1. The value of the conductance is indicated in these panels. Panels E and F show contour plot of the active to passive gain and qualify factor ($Q_{10}$), respectively, of models with the baseline MET conductance for a wide range of $r_{rl}$ and $r_{dc}$ values. $Q_{10dB}$ for models in the bottom left corner of panel F are not shown as these models are not sufficiently tuned. The solid line in E and F is the stability limit: points in the top right corner correspond to linearly unstable models.

The model described in the Methods section was used to simulate the response of the cochlea to a pure tone. Fig. 3 shows the influence of the stiffness of the DC and of the RL/PC joint on the predictions of the linear responses of the BM. The stiffnesses of the DC and of the RL/PC joint relative to the OHC stiffness are represented by the following non-dimensional parameters: $r_{dc}=K_{dc}∕K_{ohc}$ and $r_{rl}=K_{rl}∕K_{ohc}$.

Fig. 3A-D shows the BM response predicted at a basal location (20 kHz best place) by four different versions of the model. Model M1, which has very stiff DCs ($r_{dc}$ = 1000) and a very compliant RL/PC joint ($r_{rl}$ = 0.14), is nearly identical to the model used in our prior work which was based on rigid DCs (Samaras et al., 2023). The response of model M1 was calculated in a fully active case, shown in a thick solid line and representative of the *in vivo* response to a low-level stimulus, and in a passive case (obtained by setting $\epsilon_{3}$ = 0), shown in a dotted line and representative of a *postmortem* measurement. In response to a pure tone, the BM displacement predicted by the active version of M1 (shown in a solid line in Fig. 3A) has a sharp peak about 33 dB higher than the peak of the passive version of the model (shown in a dotted line), in a manner similar to the difference between the response to a low-level tone in a live animal relative to the same measurement *postmortem* (*e.g.*, Robles and Ruggero (2001)). This difference, which is illustrated in Fig. 3A, will be denoted as the active to passive gain, $G_{act∕pass}^{peak}$, throughout this discussion. Model M2 has the same RL/PC joint stiffness as M1, but has more compliant DCs ($r_{dc}$ = 5, which is similar to the value of 1 to 10 reported by Zhou et al. (2022)). Reducing the stiffness of the DC while keeping the value of $G_{hb}^{max}$ identical to the value used in model M1 significantly reduces $G_{act∕pass}^{peak}$ (see the thin solid line in Figs. 3B). Models M3 and M4 are identical to models M1 and M2, respectively, except that $r_{rl}$ increases to 12.33. This increase in $r_{rl}$ leads to a higher $G_{act∕pass}^{peak}$ compared to Ml and M2 (see the thin solid lines in Fig. 3C-D).

The influence of $r_{dc}$ and $r_{rl}$ on the active to passive gain is more systematically examined in Fig. 3E. Cross symbols correspond to the combinations of $r_{dc}$ and $r_{rl}$ values used in models M1–M4. For any value of $r_{rl}$, $G_{act∕pass}^{peak}$ decreases monotonically as $r_{dc}$ decreases. $G_{act∕pass}^{peak}$ is highly sensitive to $r_{dc}$, as indicated by the large decrease in gain as $r_{dc}$ is reduced. $G_{act∕pass}^{peak}$ is not as sensitive to $r_{rl}$, but becomes maximum when $r_{rl}$ has the value of 12.33 used in M3–M4. The boundary of linear stability is indicated by the thick solid line. Models with both high $r_{dc}$ and $r_{rl}$ values (*i.e.* in the top right corner of Fig. 3E) are linearly unstable. While the frequency response of the linearized model can be computed in the case of a linearly unstable model, this response is not physically meaningful as discussed in the Methods section. Fig. 3F shows the influence of $r_{dc}$ and $r_{rl}$ on the frequency sensitivity of the model, evaluated using the qualify factor, $Q_{10dB}$, of the active BM response. There is a broad range of parameter values that results in large $Q_{10dB}$ values with a maximum observed when $r_{rl}$ is low (< 5) and $r_{dc}$ is between 10 and 1000.

We also considered versions of models M2, M3 and M4 where the MET conductance values, $G_{hb}^{max}(x)$, were adjusted at basal locations such that $G_{act∕pass}^{peak}$ has approximately the same value as in model Ml at the 20 kHz best place. The active responses of these models are shown in thick solid lines in Fig. 3B-D. For model M3, the MET conductance was reduced to $0.89\times G_{hb,BL}^{max}$; in models M2 and M4, the MET conductance was increased to $1.70\times G_{hb,BL}^{max}$ in M2 and $1.62\times G_{hb,BL}^{max}$, respectively. These models with adjusted MET conductance were used in the remainder of this study. While the models with adjusted MET conductance have the same $G_{act∕pass}^{peak}$ value, the shape and tuning of the active model response are quite different. For example, model M2 predicts a notch at around 18 kHz which is not observed in other models. The $Q_{10dB}$ values of models M1, M2, M3 and M4 with adjusted MET conductance are 4.6, 5.2, 4.2, and 4.3, respectively, which indicates that model M2 is the most frequency selective and model M3 is the least frequency selective.

### 3.2. Nonlinear micromechanical responses of the four models

**Fig. 4.**
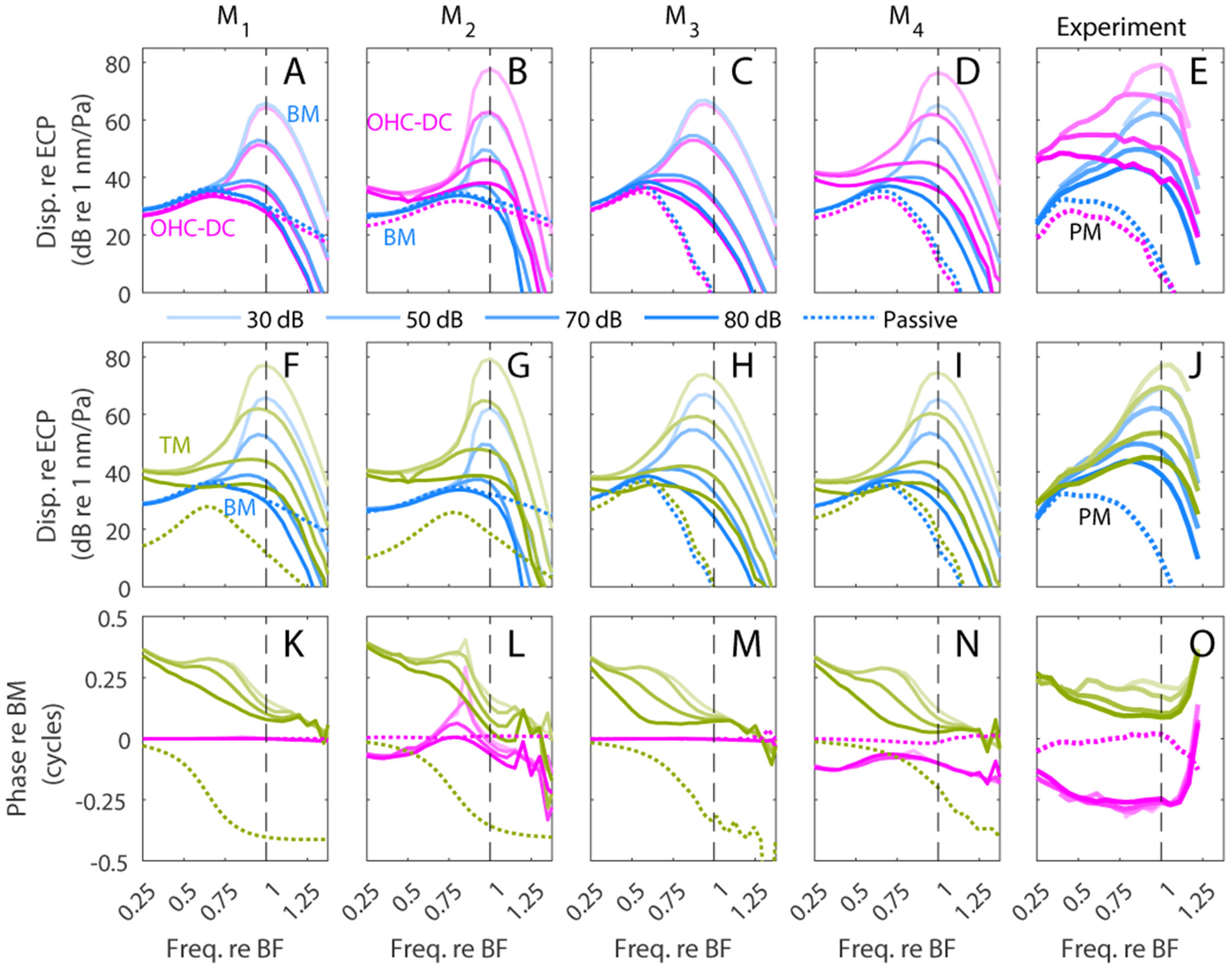
Comparison of nonlinear model predictions for OoC micromechanics to *in vivo* experiments for stimulus levels of 30, 50, 70, and 80 dB SPL. Amplitude of the displacements relative to the EC pressure are plotted at the BM (blue lines) and OHC-DC junction (magenta lines) in the 1st row; and at the BM and TM (green lines) in the 2nd row. The 3rd row shows the phase of the OHC-DC junction and TM relative to the BM. 1st column: M1; 2nd column: M2; 3rd column: M4; 4th column: M5; 5th column: experiments from Dewey et al. (2021). Model predictions are along the direction of the DC and OHCs. Experiments are in the OCT direction, which is about 30 degrees from the OHC and DC direction (see Fig. 1B in Dewey et al. (2021)). Dotted lines corresponds to passive model predictions and *postmortem* measurements.

The responses to pure tones predicted by models M1, M2, M3 and M4 and *in vivo* experimental results from Dewey et al. (2021) are shown in Fig. 4 for sound pressure levels (SPLs) between 30 dB and 80 dB. While Fig. 3 was based on the response of a linearized models, Fig. 4 shows simulations of models with nonlinear MET channels in order to predict the effect of SPL on the response. Note that the model predictions are shown at the base of the gerbil cochlea (BF = 20 kHz) while the experiments from Dewey et al. are at the apex of the mouse cochlea (BF = 9 kHz). However, the responses at the apex of the mouse cochlea have been shown to be similar to the responses at the base of other small rodents (Gao et al., 2013). To facilitate the comparison between model and experiments despite the difference in BF, the frequency is normalized to the BF.

All models predict similar BM responses as in the measurements (compare Fig. 4A-D to Fig. 4E). In all models, the compressive nonlinearity of the BM is limited to frequencies around BF and down to about 0.6× BF. The TM is predicted to vibrate several dB more than the BM at BF in response to a 30 dB SPL tone (12 dB in Fig. 4F; 17 dB in Fig. 4G; 7 dB in Fig. 4H; and 9 dB in Fig. 4I]) as in experiments (8 dB in Fig. 4J). Furthermore, the TM has a small phase lead relative to the BM that decreases with frequency, as observed experimentally (Fig. 4K-O). Experiments show that the TM has a similar frequency range of nonlinearity as the BM (Fig. 4J). The TM is predicted to have a significant nonlinearity sub-BF in models M1 and M2. When the stiffness of the RL/PC joint is increased (Model M3 and M4), the TM still has a nonlinear response sub-BF, but the size of the nonlinearity is reduced. Furthermore, the passive TM response is significantly below the passive BM response in the models with low RL/PC joint stiffness (Fig. 4F-G) but is nearly identical to the passive BM response in the models with high RL/PC joint stiffness (Fig. 4H-I). While *postmortem* TM responses are not available in data from Dewey et al. others have reported similar BM and TM *postmortem* responses (Lee et al., 2016).

There are significant differences in the predictions by the four models for the vibrations of the OHC-DC junction. In the two models with stiff DCs (M1 and M3), the displacement at the OHC-DC junction is nearly identical to the displacement of the BM (when projected onto the same direction), which is clearly inconsistent with the experimental results that show vibrations of larger amplitude at the OHC-DC junction than at the BM (Fig. 4E) and a relative phase not equal to 0 (Fig. 4O). The higher amplitude and broader frequency range of nonlinearity observed in experiments for the displacement of the OHC-DC junction are only predicted by models with significantly more compliant DCs, *i.e.* models M2 and M4 (Fig. 4B and D). In these models, the OHC-DC junction response is significantly above the response predicted by the passive model not only at BF but also at frequencies multiple octaves below BF; this is consistent with the difference between the live and *postmortem* experimental responses (Fig. 4E). However, in model M2, which has a very low RL/PC joint stiffness, the phase of the OHC-DC junction tends to lead the BM in a narrow frequency range below BF with a peak when the frequency is near 0.8× BF (Fig. 4L) in contrast to the experimental data which shows a nearly constant phase lag of about a quarter of a cycle (Fig. 4O). Model M4, which has a stiffer RL/PC joint, makes predictions for the phase of the displacement OHC-DC junction that are more in line with experiments (Fig. 4N): the DC displacement lags the BM displacement by a small amount, approximately 0.1 to 0.2 cycles, throughout the plotted frequency range.

Overall, the results of Fig. 4 show that the predictions of model M4, which has compliant DCs and a fairly stiff RL/PC joint, are the most consistent with the experimental results.

### 3.3. Quantification of the effect of OHCs on the sub-BF micromechanical response

**Fig. 5.**
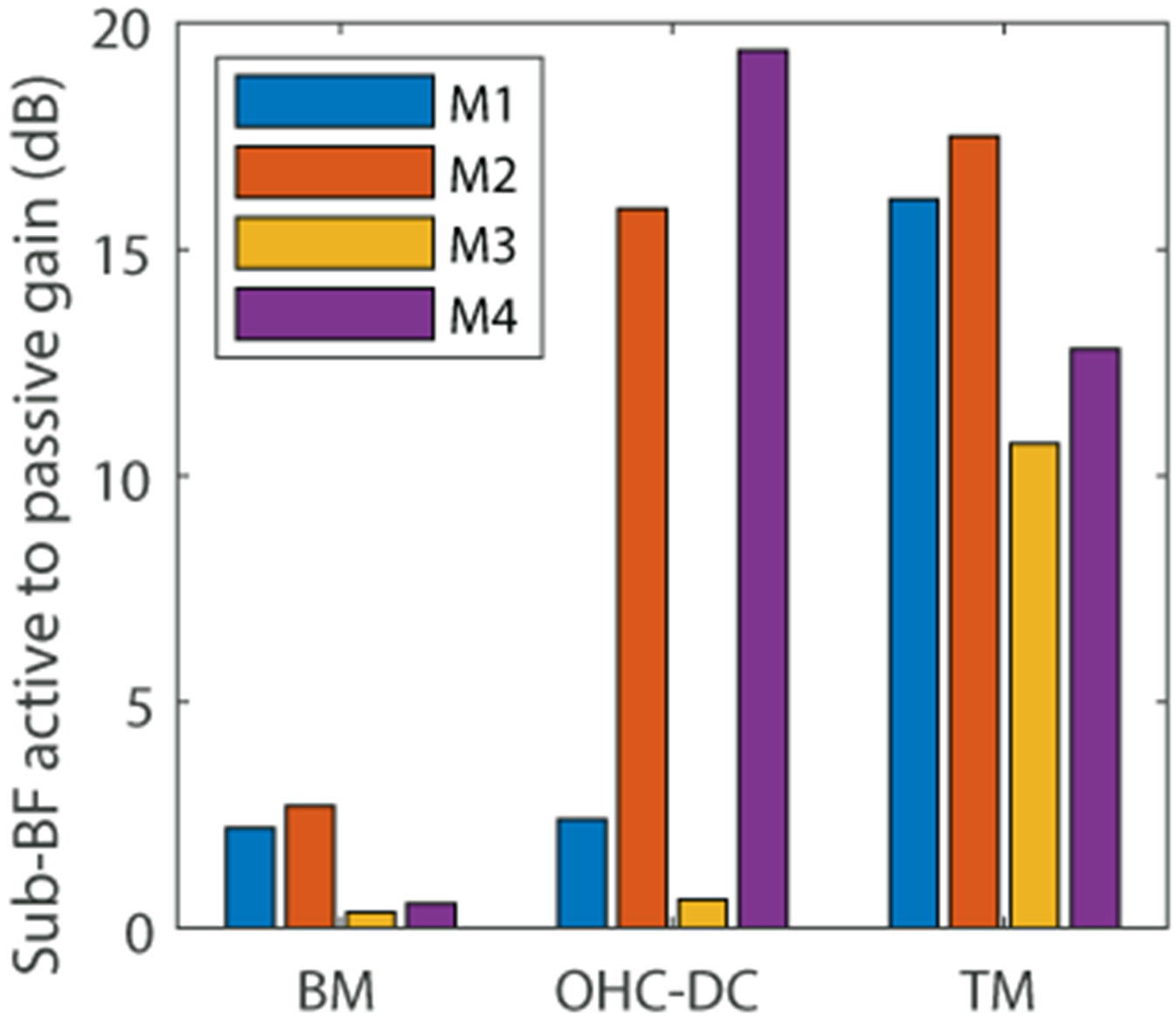
Active to passive gain of the micromechanical response on the BM, OHC/DC junction, and TM at the frequency BF/2 in models M1, M2, M3 and M4 with adjusted MET conductance.

To quantify the influence of OHCs on the micromechanical response below BF, we computed the ratio of the response predicted by the linear active models to the response predicted by passive models at a frequency of BF/2. This ratio, which we termed the sub-BF active to passive gain, was computed on the BM, OHC/DC junction, and TM, and is shown in Fig. 5. It is observed that models M1 and M2 predict small but non negligible (2 to 3 dB) active to passive gain on the BM while models M3 and M4 have negligible gain of 0.3 to 0.5 dB on the BM. The active to passive gain of the OHC/DC junction displacement is nearly identical to the gain measured on the BM sub-BF in models M1 and M3, while it is much larger (up to about 19 dB in M4) in models that have compliant DCs, *i.e.* M2 and M4. Finally, all four models predict significant sub-BF active to passive gain on the TM, with larger gain in models M1 and M2 than models M3 and M4. Note that all models predict that the transverse TM response is similar to the transverse RL response (not shown), such that this data for the sub-BF active to passive gain would look nearly identical if calculated for the RL. These results are consistent with analysis from our recent paper (Samaras et al., 2023) as increasing the stiffness of the RL to PC joint (moving from Models M1 and M2 to Models M3 and M4) tends to decrease the sub-BF gain on the RL. The predictions of significant sub-BF active to passive gain is somewhat consistent with experimental results from Ren and coworkers (He et al., 2018, 2022), who reported a large sub-BF gain of more than 20 dB of the live RL response relative to its *postmortem* response. A few studies have reported significant sub-BF nonlinearity in the TM and RL (Lee et al., 2016; Dewey et al., 2021) while others do not observe any sub-BF nonlinearity in live animals or significant changes in the sub-BF responses *postmortem* (Cho and Puria, 2022; Strimbu et al., 2024). These discrepancies may be related to the difficulty in identifying the RL in measurements and in the 3D nature of the actual vibrations. The experimental observation of significant sub-BF nonlinearity at the OHC/DC junction is more robust (*e.g.*, Dewey et al., 2021; Cho and Puria, 2022; Strimbu et al., 2024) and may be a better test of the models.

### 3.4. Tonic response

Evaluation of the tonic (direct current) responses at multiple locations within the OoC is useful as it provides information about the relative stiffness of the different components of the OoC. For this analysis, we focus on the baseline model with high DC stiffness and low RL/PC joint stiffness (model M1) and on the model that has been observed to behave the most like experiments in Fig. 4, *i.e.* model M4 which has low DC stiffness and high RL/PC joint stiffness.

**Fig. 6.**
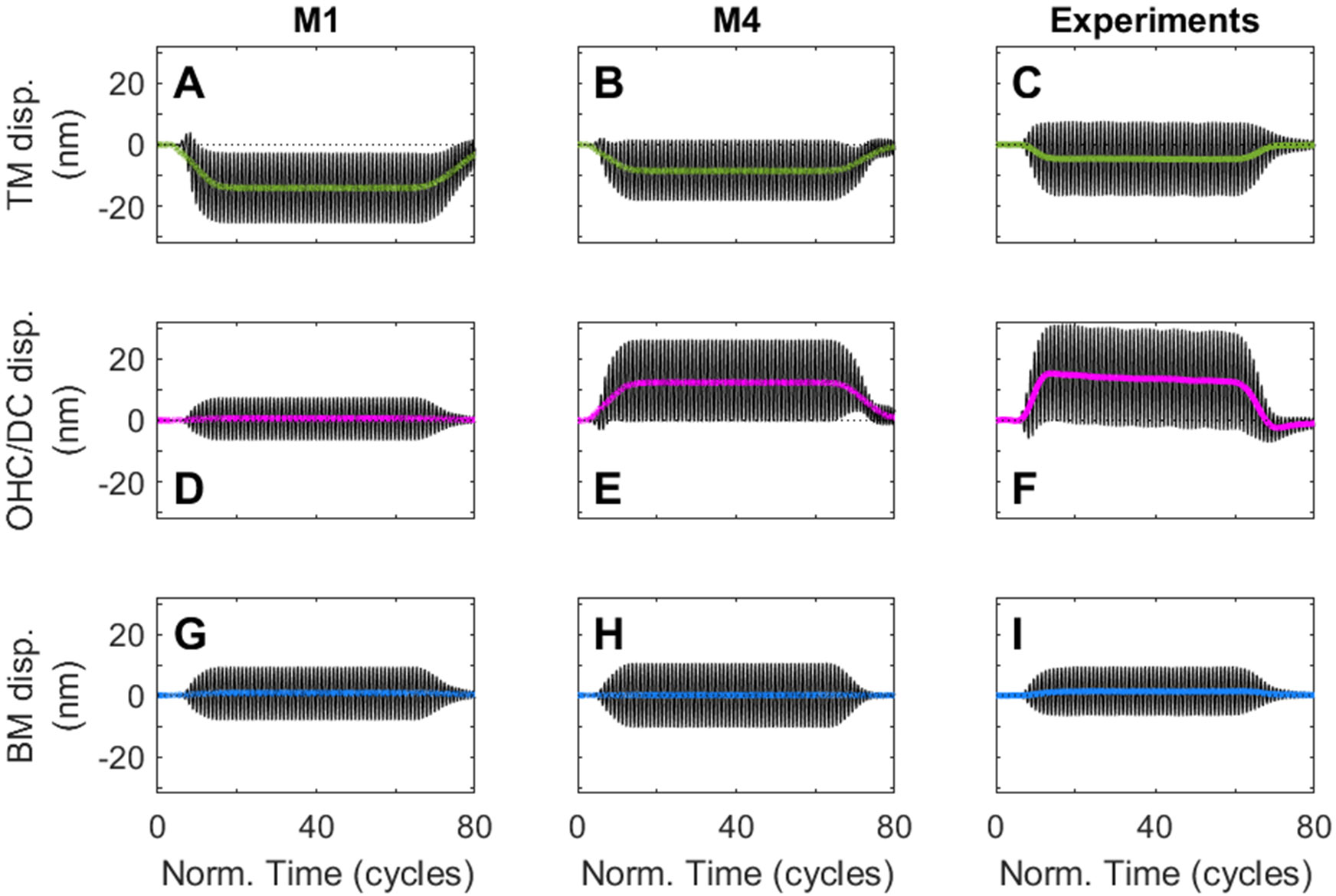
Waveform of the displacement of the TM (A–C), OHC-DC junction (D–F) and BM (G–I) in response to a pure tone. 1st column: model M1; 2nd column: model M4; 3rd column experiments from Dewey et al. (2021). The waveforms for M1 and M4 are in response to a stimulus of frequency 20 kHz and level 80 dB SPL. The waveforms from Ref. Dewey et al. (2021) are in responses to a stimulus of frequency 9 kHz and level 60 dB SPL. All waveforms are at the location of the best place. The waveforms are shown in a black solid line. The low-pass filtered data (shown in thick solid line) corresponds to the tonic displacement.

Both M1 and M4 predict that the tonic displacement of the BM is negligible (see the solid blue line in Fig. 6G-H) while the tonic displacement on the TM is significant (about 10 nm) (green solid line in Fig. 6A-B). However, only the model with compliant DCs predicts a significant tonic displacement at the OHC-DC junction (Fig. 6E). As in the experiments, the TM moves towards the scala tympani (negative tonic displacement) and the OHC-DC junction towards the scala vestibuli (positive tonic displacement), which implies that the OHC shortens. In the model, this shortening of the OHC is obtained when the resting probability of the MET channel is below 0.5 (0.3 in the results shown in the current article).

### 3.5. Analysis of force and power transfer to the BM

**Fig. 7.**
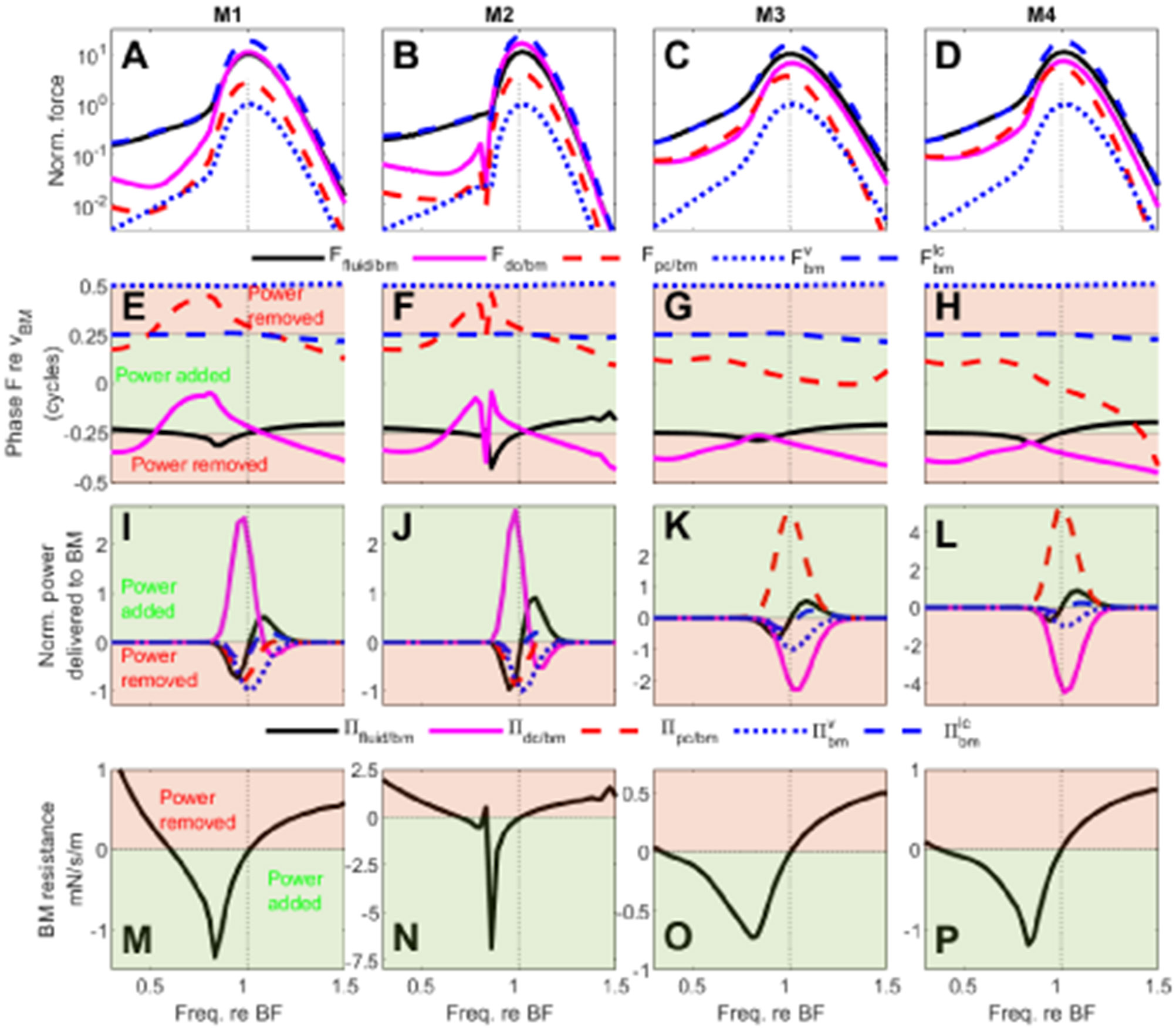
Analysis of power delivery pathways in models M1, M2, M3, and M4. Linear active models are used such that the analysis is representative of the response to low-level stimuli. 1st row: normalized magnitude of the force applied by DCs (pink line, $F_{dc∕bm}$), PCs (red line, $F_{pc∕bm}$), fluid (black line, $F_{fluid∕bm}$), viscous damping (blue dotted line, $F_{bm}^{v}$) and neighboring cross-section due to BM longitudinal coupling (blue dashed line, $F_{bm}^{lc}$) on the BM. The forces are normalized to the maximum magnitude of $F_{bm}^{v}$. 2nd row: phase of these forces relative to the BM velocity. 3rd row: power delivered to the BM by the DC, PC, and fluid. The powers due to viscous drag and longitudinal coupling on the BM are shown in blue dotted line and blue dashed lines, respectively. All powers are normalized by the peak absolute value of the power dissipated by viscous drag on the BM. 4th row: BM resistance.

Models M1, M2, M3, and M4 all predict a significant compressive nonlinearity of the BM due to the feedback from OHCs, which suggests that OHCs may deliver mechanical power to the BM. The delivery of power to the BM in response to low-level stimuli was analyzed in these four models (Fig. 7). In these models, two structural pathways may allow OoC forces and mechanical power to be transmitted to the BM: the DC pathway, *i.e.* the transmission of force by the DC, and the RL-PC pathway, *i.e.* the transmission of force by the PC (see Fig. 2).

In models M1 and M2, which have a compliant RL/PC joint, the force applied by the DC pathway, $F_{dc∕bm}$, is significantly greater than the force applied by the RL-PC pathway, $F_{pc∕bm}$ (Fig. 7A-B). Furthermore, $F_{pc∕bm}$ is out-of-phase with the BM velocity by more than a quarter cycle from ≈ 0.5× BF to BF (Fig. 7E-F), such that this force removes power from the BM (Fig. 7I-J). In the same frequency range, $F_{dc∕bm}$ is nearly in phase with the BM velocity (Fig. 7E-F) such that this force delivers power to the BM (Fig. 7I-J). Because of the combination of larger force magnitude for $F_{dc∕bm}$ and near in-phase relationship to BM velocity, more power is delivered by the DCs than the sum of what is absorbed by the PC, dissipated by viscous drag on the BM (shown in a dotted blue line in Fig. 7I-J) and absorbed by neighboring cross-sections due to BM longitudinal coupling (shown in a dashed blue line in Fig. 7I-J). As a consequence of this net addition of power, the fluid removes power from the BM in the frequency range from about 0.6× BF to BF (Fig. 7I-J). This means that the BM delivers power to the fluid and implies that the BM resistance is negative (Fig. 7M-N) in this frequency range.

In the models with a stiffer RL-PC joint (models M3 and M4), the BM still delivers power to the fluid from about 0.4×BF to BF (Fig. 7K-L and O-P). However, how this power delivery is achieved is strikingly different from what is predicted by models M1 and M2. Due to the higher RL-PC joint stiffness, $F_{dc∕bm}$ and $F_{pc∕bm}$ have a similar magnitude (Fig. 7C-D). Furthermore, $F_{pc∕bm}$ is now nearly in phase with the BM velocity while $F_{dc∕bm}$ is more than a quarter of cycle out-of-phase (Fig. 7G-H), such that $F_{pc∕bm}$ adds power to the BM and $F_{dc∕bm}$ removes power from the BM. The power added by the RL-PC pathway is larger than the sum of the power removed by the DC pathway, viscous drag and neighboring cross-sections in the frequency range that extends from about 0.4×BF to BF, which allows the BM to deliver power to the fluid.

Comparisons of the power transfer analysis for M1 and M2 (1st and 2nd columns of Fig. 7), and for M3 and M4 (3rd and 4th column of Fig. 7), show that the value of $r_{dc}$ does not significantly affect the main conclusions of this analysis. Power is delivered through the DC pathway in models with a very compliant RL/PC joint (M1 and M2) and through the RL-PC pathway in models with a stiffer RL/PC joint (M3 and M4).

### 3.6. Response of model M4 with a more realistic MET conductance function

The results so far have focused on the responses of the models at a basal location tuned to 20 kHz. Figure S3 in Supporting Information shows the nonlinear responses of these models at the 8 kHz location where the responses at the BM, OHC/DC junction and TM are similar to the 20 kHz responses shown in Fig. 4. Figure S2 in Supporting Information shows the equivalent of Fig. 3 at a more apical location tuned to 5 kHz. Some of the key trends observed in Fig. 3 are observed at this more apical location: decreasing $r_{dc}$ decreases the active to passive gain of the BM response while there is an optimal value of $r_{rl}$ that results in the maximum gain (however, this optimal value is higher than in Fig. 3 and is within the range of parameters that correspond to unstable models). The models with adjusted MET conductance recover some of the gain, but overall 1) the gains are lower than at the more basal location; 2) the tuning is significantly broader. At even more apical locations, the active to passive gain predicted by these models is very small (not shown).

**Fig. 8.**
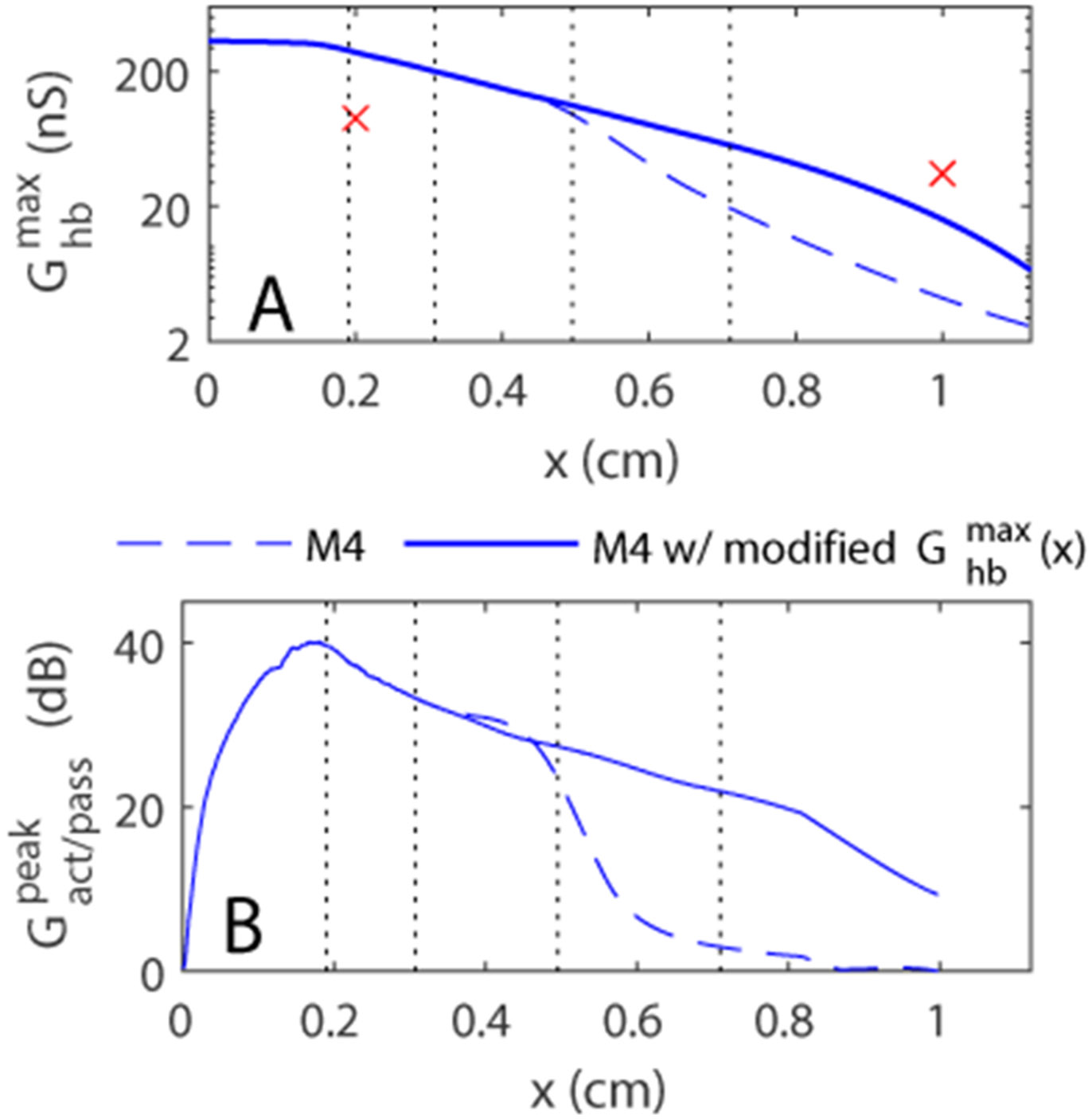
A. MET conductance for model M4 (*i.e.* $1.70\times G_{hb,BL}^{max}(x)$) and for a modified version of model M4. The red crosses correspond to the values used by Shokrian et al. (2025). B. Active to passive BM gain of the two versions of model M4. The vertical dotted lines correspond to the locations used in Fig. 9.

The lack of gain at apical locations is linked to how the MET conductance function, $G_{hb}^{max}(x)$, was chosen. The spatial variation of the conductance in the baseline model, $G_{hb,BL}^{max}(x)$, was selected to avoid a low frequency instability in some of the models (especially model M3). To simplify the analysis, the MET conductance function in models M2, M3 and M4 with adjusted MET conductance was scaled uniformly by multiplying $G_{hb,BL}^{max}(x)$ by a constant factor. With this constraint, maintaining the stability of model M3 requires us to set $G_{hb,BL}^{max}(x)$ to an artificially low value at apical locations, such that all models have a low MET conductance at these locations (see the MET conductance of model M4 with adjusted conductance in Fig. 8A). This low MET conductance at the apex makes the models essentially passive at apical locations. However, model M4 remains stable with a significantly higher MET conductance values at apical locations if $G_{hb}^{max}(x)$ is allowed to deviate from being proportional to $G_{hb,BL}^{max}(x)$. We considered a revised version of model M4 with a more realistic MET conductance function (Fig. 8A).

**Fig. 9.**
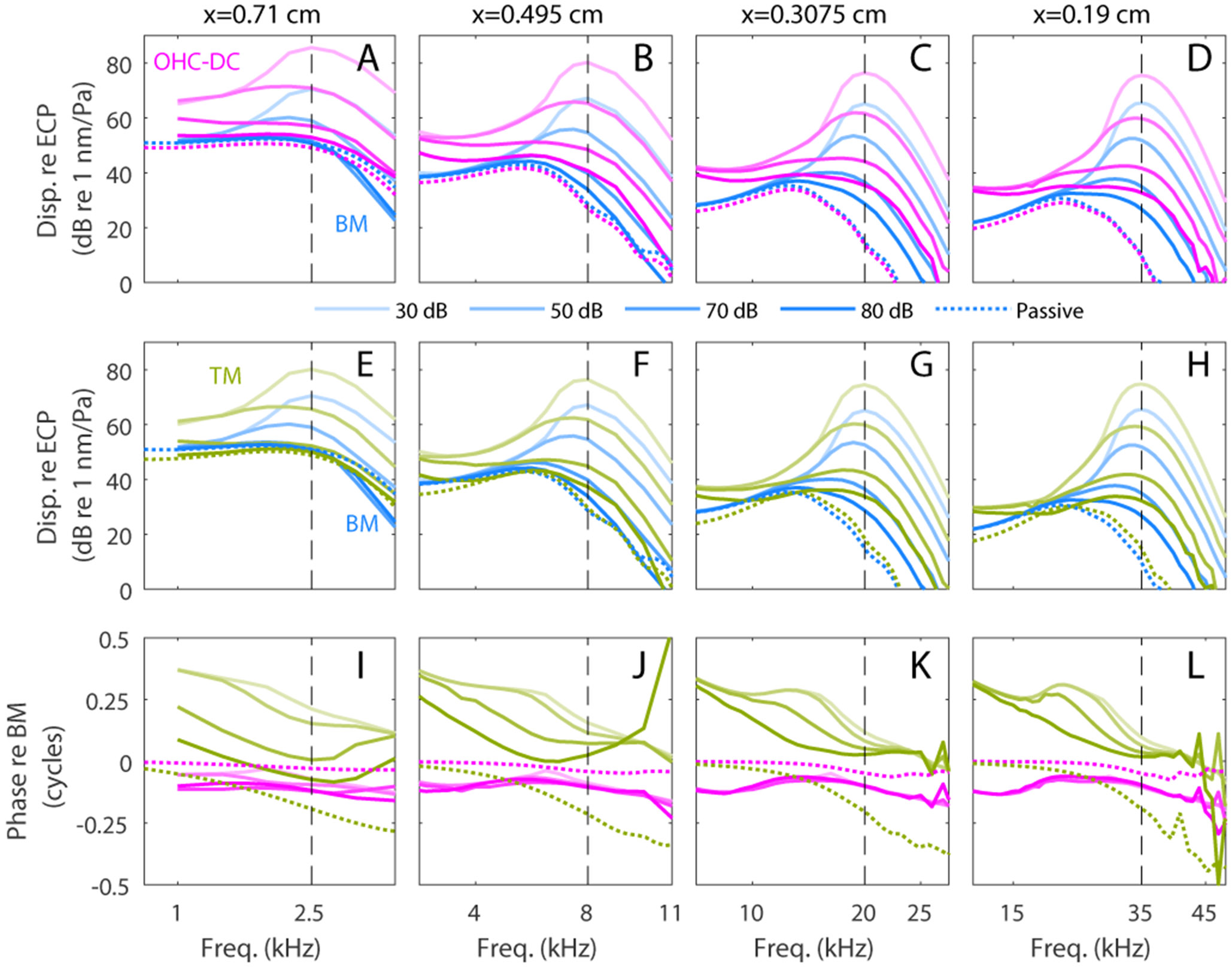
Nonlinear responses at multiple longitudinal locations predicted by model M4 with the modified MET conductance function. The responses at the BM, OHC DC junction, and TM are shown at x = 0.71 cm (CF = 2.5 kHz); x = 0.495 cm (CF = 8 kHz); x = 0.3075 cm (CF = 20 kHz) and x = 0.19 cm (CF = 35 kHz) for stimuli at 30, 50, 70 and 80 dB SPL.

This model M4 with revised MET conductance spatial variation has a significant active to passive gain throughout the cochlea, with a gain that decreases from 44 dB at x = 0.08 cm to 9.4 dB at x = 1 cm (Fig. 8B). Fig. 9 shows the nonlinear responses of this model at multiple longitudinal locations and demonstrates that the model has significant nonlinearity throughout the cochlea. The responses at the 20 kHz best place of model M4 with modified MET conductance function (3rd column of Fig. 9) are nearly identical to the response of model M4 shown in Fig. 4. The responses at both more apical locations (first two columns, tuned to 2.5 kHz and 8 kHz, respectively) and a more basal location (4th column, tuned to 35 kHz) are qualitatively similar to the 20 kHz location results: 1) the OHC/DC junction and the TM vibrate significantly more than the BM; 2) the OHC/DC junction has a broad frequency range of nonlinearity; and 3) the OHC/DC junction response lags the BM response while the TM response leads the BM response (except for 80 dB response of TM at the 2.5 kHz location).

## 4. Discussion

The results of this article demonstrate that only the model with compliant DCs and fairly stiff RL/PC joint (model M4) is able to simultaneously capture a series of experimental observations regarding the sound-evoked vibrations at the OHC-DC junction and TM: 1) the OHC-DC junction vibrates significantly more than the BM *in vivo*; 2) the motion at the OHC-DC junction lags the motion of the BM; 3) nonlinearity extends to frequencies significantly below BF at the OHC-DC junction; 4) at high level, the displacement of the OHC-DC junction includes a large tonic component towards the scala vestibuli; and 5) the passive response of the TM is similar to the passive response of the BM. While models with stiff DCs and/or compliant RL/PC joint are able to predict BM responses similar to what is observed in experiments, these models fail to capture some of these key observations for the micromechanical response at the OHC-DC junction.

It should be noted that achieving high active to passive gain of the BM response at basal locations requires the use of a higher MET saturating conductance in model M4 than in model M1. The conductance values used at the 20 kHz location in these models (122 nS and 200 nS in models M1 and M4, respectively) are within one order of magnitude of the values reported in a gerbil hemicochlea by He et al. (2004) (35 nS for a basal location). The fact that the current model requires a fairly high conductance at the base in order to achieve significant nonlinearity at basal locations is likely because the model neglects 1) HB motility; and 2) the feedforward effect linked to the longitudinal tilt of OHCs and of the phalangeal processes. Furthermore, the gradient in the MET conductance function is considerably steeper than in measurements (with a ratio from x=0.2 cm to x=1.0 cm of about 65 compared to 3 according to experimental estimates of Johnson et al. (2011)).

With model M4, it is possible to use a significantly shallower MET conductance gradient (with a conductance of about 273 nS at: x = 0.2 cm and 16 nS at the x = 1 cm location, or a ratio of 17, in model M4 with modified MET conductance function). This gradient remains steeper than in measurements of the MET conductance and than what has been used in a recent model: Shokrian et al. (2025) uses 90 nS and 27 nS at the x = 0.2 cm and x = 1 cm locations, respectively (ratio of 3.33). In our model, a fairly low conductance value is needed at x = 1.0 cm to avoid a low frequency instability that likely arises from a global resonance of the system linked to the absence of dissipation for waves that reach the helicotrema. Results from Ref. Sasmal and Grosh (2019) show that this resonance disappears (which suggests that this instability would not be an issue) if tapering of the cochlear ducts and fluid viscosity are taken into account.

Our model simulations show that reducing the value of $r_{DC}$ reduces the efficiency of the cochlear amplifier, *i.e.* a higher value of the MET conductance is needed to achieve the same BM sensitivity. It is perhaps surprising that the model seems to operate in a non-optimal region of the parameter space when the parameters are constrained based on the experimental data (*i.e.* in model M4) . However, the cochlea has other requirements in addition to high sensitivity of the BM response, including maximizing IHC stimulation, stability, structural integrity, and robustness to high level stimuli. The fact that model M4 operates in a non-optimal region of the parameter space for maximizing BM sensitivity suggests that the values of parameters may be driven by some of these other requirements.

How model M4 achieves the amplification of BM-fluid traveling waves differs from the classical cochlear amplification theory. In classical models and in models M1 and M2 with a compliant RL/PC joint, the OHCs deliver power to traveling waves in a frequency range below BF by directly transmitting force and power to the BM through the DCs. However, model M4 predicts that the DCs remove power from the BM rather than deliver power. Instead of the classical DC pathway for power delivery to cochlear traveling waves, delivery of power is achieved due to the transmission of forces from the top end of OHCs (the RL) by the PCs to the BM. While power delivery may not be directly evaluated in experiments, the phase of the OHC-DC junction relative to the BM measured in experiments appears to be consistent with the absence of power delivery through the DCs, which was recently discussed by Altoè et al. (2022). Interestingly, models M4 (and M3) predict that OHCs remove power through the DCs and deliver power rough the PCs. The net power delivered to the BM is positive below BF because the PC pathway delivers slightly more power than what is removed by the DC pathway below and up to BF: for example, the power delivered by PCs is about 30% higher than the power removed by DCs at BF.

The proposed theory for power delivery from OHCs to the BM through the RL-PC pathway differs from two alternate theories that have recently been proposed. Altoè et al. (2022) considered the DCs to be compliant viscoelastic elements, as in the current work, but hypothesized that the RL vibrations resulting from OHC electromotility deliver power to the fluid directly. Guinan (2022) proposed a conceptual model called the OoC area pump model for the amplification of traveling waves in the short-wave region. In this theory, interaction of the fluid with the RL plays a key role in cochlear amplification. Electromotile forces generated by OHCs produce longitudinal motion of the fluid within the OoC which causes local changes in the OoC area. The motion of the RL associated with this area change drives the fluid in the scala media which amplifies the fluid traveling waves. Others have also considered the interactions of the upper surface of the OoC with the cochlear fluid (Lamb and Chadwick, 2014; Cormack et al., 2015; Shokrian et al., 2025; Sisto and Moleti, 2025). Lamb and Chadwick (2014) demonstrated that two propagating modes interact with each other when both the BM and the TM are directly coupled to the fluid. Sisto and Moleti (2025) considered a single TW that interacts both with the BM and the RL due to the area deformation of the OoC. Shokrian et al. (2025) recently implemented a computational model of the cochlea in which the fluid within the OoC (the Corti fluid) is coupled to the OoC structures. They found that the Corti fluid plays a key role in the transmission of OHC forces and in the amplification of cochlear traveling waves. The current model, which only includes coupling of the fluid to the BM and does not take into account the Corti fluid and the coupling of the fluid to the upper surface of the OoC, was not designed to test these theories but to evaluate if a mechanical pathway for power delivery to cochlear traveling waves is possible in a model that accounts for recent findings regarding cochlear micromechanics (large motion at OHC/DC junction).

The fact that only the BM is directly coupled to the intracochlear fluid is a key limitation of the current study. We plan to introduce coupling between the upper surface of the OoC and the fluid in the scala media, and the coupling between the OoC and the Corti fluid, in future iterations of this model. Introducing these elements would allow us to evaluate the theories from Ref. Lamb and Chadwick (2014), Cormack et al. (2015), Shokrian et al. (2025), Sisto and Moleti (2025), Guinan (2022), Altoè et al. (2022), within the context of our modeling framework. In the current model, power amplification of traveling waves must rely on delivery of power to the BM by OoC structures. Interestingly, the observations of electrically stimulated motion of the cochlear partition in a microchamber experiment by Lin et al. (2024) are consistent with the notion that the forces applied by the PCs and DCs are out-of-phase, as proposed in this article. The antiphasic action of the DC and PC on the BM may seem counterintuitive, but the role of OHCs is not simply to boost BM vibrations, but also to maximize the stimulation of inner hair cells, which is beyond the focus of the current study. In the theory proposed in the current article, three conditions must be satisfied for efficient load transfer and power delivery to the BM via the RL-PC pathway: 1) the RL must be rigid or at least fairly stiff (no significant bending); 2) the RL/PC joint must be stiff; and 3) the PC must be stiff. Some recent experimental data may challenge these assumptions. Cho and Puria (2022) recorded *in vivo* the sound-evoked vibrations of the RL at the top of the three rows of OHCs. They observed that the displacement at the RL at the top of OHC1 (nearest to the pillar cells) is significantly less than at the top of OHC2 (middle OHC) and at the top of OHC3, which they interpreted as evidence of deformation of the RL. However, they were unable to rule out the possibility that the RL motion is the superposition of a translation in the transverse direction and of a rotation around the top of the pillar cells, which is what is assumed in the current theory. In their microchamber experiments, Lin et al. (2024) observed RL deformation in response to electrical stimulation (which stimulates the electromotility of OHC) but not in response to mechanical stimulation by fluid pressure. They also observed that 1) the PCs rotate as a rigid frame when the isolated cochlear segment is stimulated by fluid pressure; 2) the outer PC bends when the cochlear segment is stimulated by an electrical stimulus. These results suggest that the outer PCs might have a relatively low stiffness when subject to bending loads arising from electromotility. Examining whether the findings of significant PC deformation and low PC stiffness apply to an *in vivo* experiment is needed, but such experiments are challenging due to the need for high resolution and multidimensional (ideally 3D, but at least 2D) measurements in order to reliably estimate the deformation of OoC structures. Our computational framework would easily allow us to introduce deformable RL and deformable outer PCs if there is strong experimental evidence for deformation of these elements. The efficiency of cochlear amplification through the RL-PC pathway depends on the stiffness of the coupling between BM displacement and RL rotation. In the current model which assumes the PCs to be rigid structures, the stiffness of this coupling corresponds to the stiffness of the RL/PC joint. If the PCs were assumed to be structures of finite stiffness, as suggested by the data from Shokrian et al.and the RL/PC joint was assumed to be rigid, the stiffness of the coupling between BM displacement and RL rotation would correspond to the stiffness of the PCs.

In addition to the simplifying assumptions listed above, note that the current model neglects the phalangeal processes (PhPs) and the longitudinal tilt of OHCs and PhPs. In some cochlear models (Motallebzadeh et al., 2018), this longitudinal tilt plays an important role in cochlear amplification. Furthermore, it may be the cause of longitudinal motion at the OHC-DC junction (Cooper et al., 2018; Meenderink and Dong, 2022; Frost et al., 2025). Longitudinal motion is not considered in the current model which assumes that the motions of OoC structures, including the DC and OHCs, are assumed to be radial and transverse only. Modeling of longitudinal motion may be considered in future work as it may significantly influence the interpretation of experimental measurements and play an important role in cochlear amplification.

In conclusion, this study demonstrates that a model with compliant DCs and a fairly stiff RL/PC joint is able to capture a series of experimental observations regarding the micromechanics of the organ of Corti. This model makes a series of simplifying assumptions regarding OoC micromechanics and the interactions of the OoC with the intracochlear cochlear fluid. While some of these assumptions may be revisited in future work, we believe that the ability of the model in its current form to capture a wide range of observations regarding OoC micromechanics makes it a useful tool to examine cochlear function. In this model, delivery of power to cochlear traveling waves is achieved not through the DCs as classically assumed but through the PCs. These theoretical results provide new insight into how OHCs may interact with the OoC to provide the remarkable sensitivity and frequency selectivity of the mammalian cochlea.

## Supplementary Material

Supplementary informationj

Figure S3

Figure S1

Figure S2

## Data Availability

The simulation data are available at (Samaras and Meaud, 2025). The MATLAB source codes for the cochlear model are available at https://github.com/meaud-lab/cochlearModel_FiniteElement3DV5_v1_0.

## Appendix A. Supplementary data

Supplementary material related to this article can be found online at https://doi.org/10.1016/j.heares.2026.109618.
