# Supplementary material for: An alternative pathway for delivery of power by outer hair cells to cochlear traveling waves
Source: Hear Res. Author manuscript; Available in PMC 2026 Jun 5. (PMC13237755; doi:10.1016/j.heares.2026.109618)
Supplement: Supplementary informationj [file NIHMS2174663-supplement-Supplementary_informationj.pdf]

Supplemental Material:

An alternative pathway for delivery of power by OHCs to cochlear traveling waves

George Samaras and Julien Meaud[a)]

George W. Woodruff School of Mechanical Engineering, Georgia Institute of Technology, 771 Ferst Drive, Atlanta, Georgia 30332, USA.

(Dated: 12 March 2026)

[a)]Corresponding author: julien.meaud@me.gatech.edu

I. GEOMETRY OF OOC MODEL

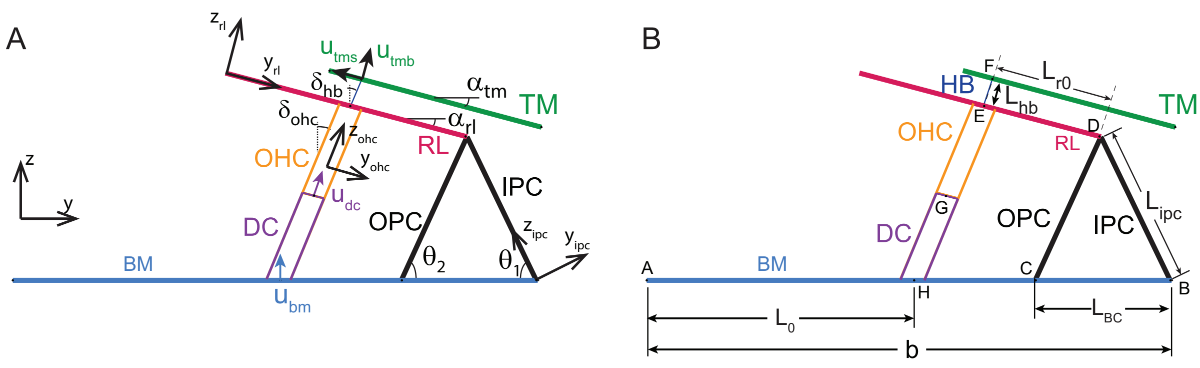

FIG. S1. Schematics of OoC model. A. Orientation of OoC structure and of the coordinate systems. B. Length of OoC structures.

x, *y*, and *z* correspond to the longitudinal, radial and transverse coordinates, respectively. The value of the geometrical parameters of the OoC model are listed in Table S1.

II. KINEMATICS AND DEFORMATION OF SPRINGS AND VISCOUS ELEMENTS

The displacement vector of the TM at point *F* is the following:

$$\vec{u}_{tm} = -u_{tms}\vec{y}_{rl} + u_{tmb}\vec{z}_{rl}. \tag{S1}$$

Because the PCs are assumed to be rigid, the displacement vector at the top of tunnel of Corti (point D) is given by:

$$\vec{u}_{ap} = D_1 U_{bm}\vec{y}_{ipc}, \tag{S2}$$

where

$$D_1 = \psi_{bm}(\frac{b}{2} - L_{BC})\frac{\sin(\theta_1+\theta_2)}{\sin\theta_2}. \tag{S3}$$

This can be used to calculate the angular deflection of the PCs relative to a fixed frame in a counterclockwise direction:

$$\Delta\theta_{pc} = -\frac{1}{L_{ipc}}D_1 U_{bm}. \tag{S4}$$

Since the RL is assumed to be a rigid body, the displacement vector at the RL at point E can be written as:

$$\vec{u}_{rl} = \vec{u}_{ap} - \Delta\theta_{rl}L_{r0}\vec{z}_{rl}, \tag{S5}$$

TABLE S1. Geometrical parameters (parameters are assumed to vary linearly with x). All angles are expressed in degrees. All lengths are expressed in μm.

Variable	Description	Value (base to apex)
θ_1	angle between between the inner PC and the BM	60
θ_2	angle between between the outer PC and the BM	60
$\alpha = \alpha_{rl} = \alpha_{tm}$	angle between radial direction and the RL and between radial direction and TM	14.75 to 25.54
δ_{ohc}	angle between OHC and transverse direction and between DC and transverse direction	$= \alpha$
δ_{hb}	angle between HB and transverse direction	$= \alpha$
L_{pc}	length of inner PC	58.324 to 100.35
L_{r0}	distance between top of PCs (point D) and top of OHC (point E)	25.092 to 49.92
L_{hb}	HB length	1 to 6
b	BM width	208.157 to 320.2
L_0	Distance between left end of BM (point A) and bottom of DC (point H)	$b(x)/2$

where $\Delta\theta_{rl}$ is angular deflection of the RL relative to a fixed frame in the counterclockwise direction. Because the HBs are assumed to be rigid bodies, the displacement vector at the RL at point E can also be written as:

$$\vec{u}_{rl} = \vec{u}_{tm} + \Delta\theta_{hb} L_{hb} \vec{y}_{rl}, \tag{S6}$$

where $\Delta\theta_{hb}$ is the rotation of the HB relative to a fixed frame. Equating Eqs. S5 and S6 can be used to express $\Delta\theta_{rl}$ and $\Delta\theta_{hb}$ as a function of the local DOFs:

$$\Delta\theta_{rl} = -\frac{1}{L_{r0}} \Big[B_{21} U_{bm} + u_{tmb} \Big], \tag{S7}$$

$$\Delta\theta_{hb} = \frac{1}{L_{hb}} \Big[A_{21} U_{bm} + u_{tms} \Big], \tag{S8}$$

where

$$A_{21} = D_1 \vec{y}_{ipc} \cdot \vec{y}_{rl} = D_1 \sin(\theta_1 - \alpha), \tag{S9}$$

$$B_{21} = -D_1 \vec{y}_{ipc} \cdot \vec{z}_{rl} = -D_1 \cos(\theta_1 - \alpha). \tag{S10}$$

Eq. S6 is used to express $\vec{u}_{rl}$ as a function of the local DOFs:

$$\vec{u}_{rl} = A_{21} U_{bm} \vec{y}_{rl} + u_{tmb} \vec{z}_{rl}. \tag{S11}$$

The deformations of the four springs in the local OoC model are given by:

$$\Delta u_{hb/rl} = (\Delta\theta_{hb} - \Delta\theta_{rl}) L_{hb}, \tag{S12}$$

$$\Delta u_{pc/rl} = (\Delta\theta_{pc} - \Delta\theta_{rl}) L_{r0}, \tag{S13}$$

$$\Delta u_{ohc}^{comp} = (\vec{u}_{dc} - \vec{u}_{rl}) \cdot \vec{z}_{ohc}, \tag{S14}$$

$$\Delta u_{dc}^{comp} = (\vec{U}_{bm} - \vec{u}_{dc}) \cdot \vec{z}_{ohc}, \tag{S15}$$

where both $\Delta u_{hb/rl}$ and $\Delta u_{pc/rl}$ are defined to be positive for counterclockwise rotation (such that $\Delta u_{hb/rl} > 0$ corresponds to the excitatory direction). These expressions can be written as:

$$\mathbf{u}_{def} = \mathbf{G^e} \mathbf{u}_{dof}, \tag{S16}$$

where $\mathbf{u}_{def} = \left[\Delta u_{hb/rl}, \Delta u_{pc/rl}, \Delta u_{ohc}^{comp}, \Delta u_{dc}^{comp}\right]^T$, $\mathbf{u}_{dof} = \left[U_{bm}, u_{tms}, u_{tmb}, u_{dc}\right]^T$, and $\mathbf{G^e}$ is the following 4×4 matrix:

$$\mathbf{G^e} = \begin{bmatrix} G_{11}^e & G_{12}^e & G_{13}^e & G_{14}^e \\ G_{21}^e & G_{22}^e & G_{23}^e & G_{24}^e \\ G_{31}^e & G_{32}^e & G_{33}^e & G_{34}^e \\ G_{41}^e & G_{42}^e & G_{43}^e & G_{44}^e \end{bmatrix} = \begin{bmatrix} G_{11}^e & 1 & G_{13}^e & 0 \\ G_{21}^e & 0 & 1 & 0 \\ G_{31}^e & 0 & G_{33}^e & 1 \\ G_{41}^e & 0 & 0 & -1 \end{bmatrix}, \tag{S17}$$

where

$$G_{11}^e = A_{21} + B_{21} \frac{L_{hb}}{L_{r0}}, \tag{S18}$$

$$G_{13}^e = \frac{L_{hb}}{L_{r0}}, \tag{S19}$$

$$G_{21}^e = B_{21} - D_1 \frac{L_{r0}}{L_{ipc}}, \tag{S20}$$

$$G_{31}^e = -A_{21} \vec{y}_{rl} \cdot \vec{z}_{ohc} = A_{21} \sin(\alpha - \delta_{ohc}), \tag{S21}$$

$$G_{33}^e = -\vec{z}_{rl} \cdot \vec{z}_{ohc} = -\cos(\alpha - \delta_{ohc}) \tag{S22}$$

$$G_{41}^e = \psi_{bm} (L_0 - \frac{L}{2}) \cos\delta_{ohc}. \tag{S23}$$

The red and magenta colors in Eq. S17 identify the kinematic coupling terms that link the deformation of the elastic elements within the OoC to the BM displacement: the red color corresponds to terms that arise because the PCs are rigidly coupled to the BM; the magenta color corresponds to terms that arise because the bottom ends of DCs are rigidly coupled to the BM.

In addition, to calculate the force applied by viscous damping in the subtectorial space, the expression for the shear displacement of the TM relative to the RL is needed. This displacement is denoted Δu_{sts} and is given by:

$$\begin{aligned}\Delta u_{sts} &= -(\vec{u}_{tm} - \vec{u}_{rl}) \cdot \vec{y}_{rl} \\ &= A_{21} U_{bm} + u_{tms}.\end{aligned} \tag{S24}$$

The two viscous elements within the organ of Corti are the subtectorial space and the DCs. Writing the velocity of these elements as the vector $\mathbf{v}_{def} = [\Delta \dot{u}_{sts}, \Delta \dot{u}_{ohc}^{comp}, \Delta \dot{u}_{dc}^{comp}]^T$, we can write:

$$\mathbf{v}_{def} = \mathbf{G}^{\mathbf{v}} \dot{\mathbf{u}}_{dof}, \tag{S25}$$

where $\mathbf{G}^{\mathbf{v}}$ is the following 3x4 matrix:

$$\mathbf{G}^{\mathbf{v}} = \begin{bmatrix} G_{11}^{v} & G_{12}^{v} & G_{13}^{v} & G_{14}^{v} \\ G_{21}^{v} & G_{22}^{v} & G_{23}^{v} & G_{24}^{v} \\ G_{31}^{v} & G_{32}^{v} & G_{33}^{v} & G_{34}^{v} \end{bmatrix} = \begin{bmatrix} A_{21} & 1 & 0 & 0 \\ G_{31}^{e} & 0 & G_{33}^{e} & 1 \\ G_{41}^{e} & 0 & 0 & -1 \end{bmatrix}. \tag{S26}$$

As in Eq. S17, the red and magenta colors in Eq. S26 identify the terms that arise due to the rigid coupling between the PCs and the BM and and between the bottom ends of the DCs and the BM, respectively.

III. EQUATIONS OF MOTION

A. For the BM

The BM is modeled as an orthotropic plate[1] with the following equation of motion:

$$\begin{aligned}\rho_{bm} h_{bm} \ddot{u}_{bm}(x,y) = &-\frac{2}{b} C_{bm} \dot{u}_{bm}(x,y) - \mathscr{L}_s \Big[u_{bm}(x,y) \Big] \\ &+ P_{f/bm}(x,y) + P_{OoC/bm}^{e}(x,y) + P_{OoC/bm}^{v}(x,y) + P_{ohc/bm}^{act}(x,y),\end{aligned} \tag{S27}$$

where ρ_{bm} is the mass density of the BM; h_{bm} is the BM thickness; C_{bm} is a damping coefficient; $P_{OoC/bm}^{e}(x,y)$, $P_{OoC/bm}^{v}(x,y)$, $P_{ohc/bm}^{act}(x,y)$ are the pressure applied by elastic element within the

OoC, by viscous elements within the OoC, and by the electromotile OHC force, respectively; $P_{f/bm}(x,y)$ is the pressure applied by the fluid on the BM:

$$P_{f/bm}(x,y) = P(x,y,z=0^-) - P(x,y,z=0^+), \tag{S28}$$

and $\mathscr{L}_s$ is the structural operator for an orthotropic plate with varying properties:

$$\begin{aligned}\mathscr{L}_s\left[u_{bm}(x,y)\right] = \frac{\partial^2}{\partial x^2}\left[D_{xx}\frac{\partial^2 u_{bm}}{\partial x^2}\right] + \frac{\partial^2}{\partial x^2}\left[D_{xy}\frac{\partial^2 u_{bm}}{\partial y^2}\right] + \frac{\partial^2}{\partial y^2}\left[D_{xy}\frac{\partial^2 u_{bm}}{\partial x^2}\right] + \\ 2\frac{\partial^2}{\partial x \partial y}\left[D_s\frac{\partial^2 u_{bm}}{\partial x \partial y}\right] + \frac{\partial^2}{\partial y^2}\left[D_{yy}\frac{\partial^2 u_{bm}}{\partial y^2}\right],\end{aligned} \tag{S29}$$

where D_{xx}, D_{xy}, D_s and D_{yy} are the bending rigidities of the orthotropic plate. Eq. S27 is projected onto the BM mode shape, $\psi_{bm}(x,y)$, to obtain the following equation:

$$\begin{aligned}M_{bm}\ddot{U}_{bm} = -C_{bm}\dot{U}_{bm} - K_{bm}U_{bm} - \mathscr{L}_{bm,2.5D}\left[U_{bm}(x)\right] \\ + F^{e}_{OoC/bm} + F^{v}_{OoC/bm} + F^{act}_{ohc/bm} + F_{f/bm},\end{aligned} \tag{S30}$$

where

$$K_{bm} = \frac{\pi^4}{2b^3}D_{yy}, \tag{S31}$$

$$M_{bm} = \rho_{bm}h_{bm}\frac{b}{2}, \tag{S32}$$

$$\begin{aligned}\mathscr{L}_{bm,2.5D}\left[U_{bm}(x)\right] = \int_{y=-b/2}^{b/2} \psi_{bm}(x,y)\Bigg[\frac{\partial^2}{\partial x^2}\left[D_{xx}\frac{\partial^2 u_{bm}}{\partial x^2}\right] \\ + \frac{\partial^2}{\partial x^2}\left[D_{xy}\frac{\partial^2 u_{bm}}{\partial y^2}\right] + \frac{\partial^2}{\partial y^2}\left[D_{xy}\frac{\partial^2 u_{bm}}{\partial x^2}\right] + \\ 2\frac{\partial^2}{\partial x \partial y}\left[D_s\frac{\partial^2 u_{bm}}{\partial x \partial y}\right]\Bigg]dy,\end{aligned} \tag{S33}$$

$$F_{f/bm} = \int_{y=-b/2}^{b/2} \psi_{bm}(x,y)P_{f/bm}(x,y)dy. \tag{S34}$$

$F^{e}_{OoC/bm}$, $F^{v}_{OoC/bm}$, and $F^{act}_{ohc/bm}$ are the generalized forces applied elastic elements within the OoC, by viscous elements within the OoC, and by OHC electromotility, respectively.

B. For the TM

The equations of motion for the radial and bending motion of the TM are the following[1]:

$$\begin{aligned} M_{tms}\ddot{u}_{tms} &= -C_{tms}\dot{u}_{tms} - K_{tms}u_{tms} - \mathscr{L}_{tms}\Big[u_{tms}(x), \dot{u}_{tms}(x)\Big] \\ &\quad + F^{e}_{OoC/tms} + F^{v}_{OoC/tms} + F^{act}_{ohc/tms}, \end{aligned} \tag{S35}$$

$$\begin{aligned} M_{tmb}\ddot{u}_{tmb} &= -C_{tmb}\dot{u}_{tmb} - K_{tmb}u_{tmb} - \mathscr{L}_{tmb}\Big[u_{tmb}(x), \dot{u}_{tmb}(x)\Big] \\ &\quad + F^{e}_{OoC/tmb} + F^{v}_{OoC/tmb} + F^{act}_{ohc/tmb}, \end{aligned} \tag{S36}$$

where K_{tms} and K_{tmb} are the stiffnesses in the shearing and bending directions of the attachment of the TM to the spiral limbus; C_{tms} and C_{tmb} are the viscous damping coefficients of the TM in the shearing and bending directions; M_{tms} and M_{tmb} are the effective masses of the TM in the shearing and bending directions; $F^{e}_{OoC/tmj}$, $F^{v}_{OoC/tmj}$ and $F^{act}_{ohc/tmj}$ (where $j = s$ and $j = b$) are the generalized forced applied on the TM by elastic elements within the OoC, by viscous elements within the OoC and by OHC electromotility, respectively; $\mathscr{L}_{tms}$ and $\mathscr{L}_{tmb}$ are the following operators for TM viscoelastic longitudinal coupling:

$$\mathscr{L}_{tms}\Big[u_{tms}(x), \dot{u}_{tms}(x)\Big] = -\frac{\partial}{\partial x}\left[A_{tms}G_{tm}\frac{\partial u_{tms}}{\partial x} + A_{tms}\eta_{tm}\frac{\partial \dot{u}_{tms}}{\partial x}\right], \tag{S37}$$

$$\mathscr{L}_{tmb}\Big[u_{tmb}(x), \dot{u}_{tmb}(x)\Big] = -\frac{\partial}{\partial x}\left[A_{tmb}G_{tm}\frac{\partial u_{tm}}{\partial x} + A_{tmb}\eta_{tm}\frac{\partial \dot{u}_{tmb}}{\partial x}\right], \tag{S38}$$

where A_{tms} and A_{tmb} are the effective area of the TM cross-section in shearing and bending directions, respectively; G_{tm} and η_{tm} are the shear modulus and shear viscosity of the TM, respectively.

C. For DCs

The equation of motion for the DC is given by:

$$M_{dc}\ddot{u}_{dc} = F^{e}_{OoC/dc} + F^{v}_{OoC/dc} + F^{act}_{ohc/dc}, \tag{S39}$$

where M_{dc} is the mass of the DC; $F^{e}_{OoC/dc}$, $F^{v}_{OoC/dc}$ and $F^{act}_{ohc/dc}$ are the generalized forced applied on the DC by elastic elements within the OoC, by viscous elements within the OoC and by OHC electromotility, respectively.

D. Overall equations

Equations S27, S35, S36 and S39 can be combined into a single system of 2nd order ODEs in matrix-vector form:

$$\mathbf{M}_{loc}\ddot{\mathbf{u}}_{dof} = -\mathbf{C}_{loc}\dot{\mathbf{u}}_{dof} - \mathbf{K}_{loc}\mathbf{u}_{dof} - \mathscr{L}\left[\mathbf{u}_{dof}, \dot{\mathbf{u}}_{dof}\right] + \mathbf{F}^{e}_{OoC/dof} + \mathbf{F}^{v}_{OoC/dof} + \mathbf{F}^{act}_{ohc/dof} + \mathbf{F}_{f}, \tag{S40}$$

where

$$\mathbf{M}_{loc} = \begin{bmatrix} M_{bm} & 0 & 0 & 0 \\ 0 & M_{tms} & 0 & 0 \\ 0 & 0 & M_{tmb} & 0 \\ 0 & 0 & 0 & M_{dc} \end{bmatrix}, \tag{S41}$$

$$\mathbf{C}_{loc} = \begin{bmatrix} C_{bm} & 0 & 0 & 0 \\ 0 & C_{tms} & 0 & 0 \\ 0 & 0 & C_{tmb} & 0 \\ 0 & 0 & 0 & 0 \end{bmatrix}, \tag{S42}$$

$$\mathbf{K}_{loc} = \begin{bmatrix} K_{bm} & 0 & 0 & 0 \\ 0 & K_{tms} & 0 & 0 \\ 0 & 0 & K_{tmb} & 0 \\ 0 & 0 & 0 & 0 \end{bmatrix}, \tag{S43}$$

$$\mathscr{L}\left[\mathbf{u}_{dof}, \dot{\mathbf{u}}_{dof}\right] = \begin{bmatrix} \mathscr{L}_{bm,2.5D}\left[u_{bm}(x,y)\right] \\ \mathscr{L}_{tms}\left[u_{tms}(x), \dot{u}_{tms}(x)\right] \\ \mathscr{L}_{tmb}\left[u_{tmb}(x), \dot{u}_{tmb}(x)\right] \\ 0 \end{bmatrix}, \tag{S44}$$

$$\mathbf{F}_{f} = \begin{bmatrix} F_{f/bm} \\ 0 \\ 0 \\ 0 \end{bmatrix}. \tag{S45}$$

The expression for $\mathbf{F}^{e}_{OoC/dof}$ can be found by evaluating the potential energy density due to the elastic elements internal to the OoC (OHC, DC, RL, and HB):

$$v_{OoC}(x) = v_{hb}\left[\mathbf{u}_{dof}(x)\right] + v_{rl}\left[\mathbf{u}_{dof}(x)\right] + v_{ohc}\left[\mathbf{u}_{dof}(x)\right] + v_{dc}\left[\mathbf{u}_{dof}(x)\right], \tag{S46}$$

where the expression for the energy densities of these elements is found in Eqs. (3-6). The force vector applied by these elements on local DOFs is given by:

$$\mathbf{F}^{e}_{OoC/dof} = -\frac{\partial v_{OoC}}{\partial \mathbf{u}_{dof}}, \tag{S47}$$

which can be expressed as:

$$\mathbf{F}^{e}_{OoC} = -\mathbf{K}_{OoC}\mathbf{u}_{dof}, \tag{S48}$$

where

$$\mathbf{K}_{OoC} = \mathbf{G}^{\mathbf{e}T}\mathbf{K}_{def}\mathbf{G}^{\mathbf{e}}, \tag{S49}$$

where

$$\mathbf{K}_{def} = \begin{bmatrix} N_{ohc}K_{hb} & 0 & 0 & 0 \\ 0 & K_{rl} & 0 & 0 \\ 0 & 0 & N_{ohc}K_{ohc} & 0 \\ 0 & 0 & 0 & N_{ohc}K_{dc} \end{bmatrix}. \tag{S50}$$

Similarly, the generalized work done by viscous forces within the OoC can be written as:

$$Q^{viscous}_{Ooc} = Q^{v}_{sts}(x) + Q^{v}_{dc}(x), \tag{S51}$$

where

$$Q^{v}_{sts}(x) = -C_{sts} \cdot \Delta\dot{u}_{sts} \cdot \Delta u_{sts}, \tag{S52}$$

$$Q^{v}_{ohc}(x) = -C_{ohc} \cdot \Delta\dot{u}_{ohc} \cdot \Delta u_{ohc}, \tag{S53}$$

$$Q^{v}_{dc}(x) = -C_{dc} \cdot \Delta\dot{u}^{comp}_{dc} \cdot \Delta u^{comp}_{dc}. \tag{S54}$$

The force applied by these viscous elements on DOFs is given by:

$$\mathbf{F}^{v}_{OoC/dof} = \frac{\partial Q^{v}_{Ooc}}{\partial \mathbf{u}_{dof}}, \tag{S55}$$

which can be expressed as:

$$\mathbf{F}^{v}_{OoC} = -\mathbf{C}_{OoC}\dot{\mathbf{u}}_{dof}, \tag{S56}$$

where

$$\mathbf{C}_{OoC} = \mathbf{G}^{\mathbf{v}T}\mathbf{C}_{def}\mathbf{G}^{\mathbf{v}}, \tag{S57}$$

where

$$\mathbf{C}_{def} = \begin{bmatrix} C_{st} & 0 & 0 \\ 0 & C_{ohc} & 0 \\ 0 & 0 & C_{dc} \end{bmatrix}. \tag{S58}$$

The generalized work done by electromotility is the following:

$$Q_{ohc}^{act} = f_{ohc}^{act} \Delta u_{ohc}^{comp}. \tag{S59}$$

This expression can be used to derive the expression for the force $\mathbf{F}_{ohc/dof}^{act}$ applied by electromotility on the DOFs:

$$\mathbf{F}_{ohc/dof}^{act} = \frac{\partial Q_{ohc}^{act}}{\partial \vec{u}_{dof}}, \tag{S60}$$

which yields

$$\mathbf{F}_{ohc/dof}^{act} = \begin{bmatrix} G_{31}^{e} \\ G_{32}^{e} \\ G_{33}^{e} \\ G_{34}^{e} \end{bmatrix} f_{ohc}^{act}. \tag{S61}$$

These equations can be used to write the equation of motion of the OoC as the following system of PDEs:

$$\mathbf{M}_{loc}\ddot{\mathbf{u}}_{dof} + \left[\mathbf{C}_{loc} + \mathbf{C}_{OoC}\right]\dot{\mathbf{u}}_{dof} + \left[\mathbf{K}_{loc} + \mathbf{K}_{OoC}\right]\mathbf{u}_{dof} + \mathscr{L}\left[\mathbf{u}_{dof}, \dot{\mathbf{u}}_{dof}\right] = \mathbf{F}_{ohc/dof}^{act} + \mathbf{F}_{f}. \tag{S62}$$

As described in previous work[1–3], the system of PDEs for the motion of the OoC structures, along with the system of PDEs for the electrical potential and the fluid pressure inside the ducts are used to derive a system of ODEs for the overall dynamics of the cochlear model based on the finite element method. This system is solved numerically using a Runge Kutta method (ode45 in MATLAB).

IV. CALCULATION OF THE FORCES APPLIED BY THE DCS AND PCS ON THE BM

The DCs apply forces directly on the BM due to the rigid coupling between the bottom end of the DCs and the BM. The total force applied by the DCs on the BM is the sum of an elastic force, $F_{dc/bm}^{e}$ and of a viscous force, $F_{dc/bm}^{v}$:

$$F_{dc/bm} = F_{dc/bm}^{e} + F_{dc/bm}^{v}. \tag{S63}$$

Calculation of the force applied by the PCs on the BM, $F_{pc/bm}$ is more complicated as $F_{pc/bm}$ is linked to the assumption of rigid coupling between the PCs and the BM. This rigid coupling gives rise to kinematic coupling between elements of the OoC (HBs, OHCs, RL and STS) and the BM

(*i.e.* $G^e_{i1} \neq 0$ for $i = 1,.., 3$ and $G^v_{i1} \neq 0$ for $i = 1,.., 2$). This implies that these springs and viscous elements apply generalized forces on the BM. The total force applied by the PCs on the BM is given by:

$$F_{pc/bm} = F^e_{hb/bm} + F^e_{rl/bm} + F^e_{ohc/bm} + F^v_{ohc/bm} + F^v_{sts/bm} + F^{act}_{ohc/bm}, \tag{S64}$$

where $F^e_{dc/bm}$, $F^e_{hb/bm}$, $F^e_{rl/bm}$, $F^e_{ohc/bm}$, and $F^e_{dc/bm}$ are the elastic forces applied by the DC, HB, RL/PC joint and OHC, respectively; $F^v_{dc/bm}$, $F^v_{ohc/bm}$ and $F^v_{sts/bm}$ are the viscous forces applied the DC, OHC, and STS, respectively; and $F^{act}_{ohc/bm}$ is the active OHC force applied on the BM.

Equation S48 yields the following expression for the elastic forces applied on the BM:

$$F^e_{hb/bm} = -N_{ohc} K_{hb} G^e_{11} \sum_{j=1}^{4} G^e_{1j} u_{dof,j}, \tag{S65}$$

$$F^e_{rl/bm} = -K_{rl} G^e_{21} \sum_{j=1}^{4} G^e_{2j} u_{dof,j}, \tag{S66}$$

$$F^e_{ohc/bm} = -N_{ohc} K_{ohc} G^e_{31} \sum_{j=1}^{4} G^e_{3j} u_{dof,j}, \tag{S67}$$

$$F^e_{dc/bm} = -N_{ohc} K_{dc} G^e_{41} \sum_{j=1}^{4} G^e_{4j} u_{dof,j},. \tag{S68}$$

Equation S55 yields the following expression for the viscous forces applied on the BM:

$$F^v_{sts/bm} = -C_{sts} G^v_{11} \sum_{j=1}^{4} G^v_{1V} \dot{u}_{dof,j} \tag{S69}$$

$$F^v_{ohc/bm} = -N_{ohc} C_{ohc} G^v_{21} \sum_{j=1}^{4} G^v_{2j} \dot{u}_{dof,j}, \tag{S70}$$

$$F^v_{dc/bm} = -N_{ohc} C_{dc} G^v_{31} \sum_{j=1}^{4} G^v_{3j} \dot{u}_{dof,j},. \tag{S71}$$

Equation S61 yields the following expression for the force applied by electromotility on the BM:

$$F^{act}_{ohc/bm} = G^e_{31} f^{act}_{ohc}. \tag{S72}$$

Since the forces $F^e_{hb/bm}$, $F^e_{rl/bm}$, $F^e_{ohc/bm}$, $F^v_{sts/bm}$ and $F^v_{ohc/bm}$ are non-zero due to the kinematic coupling terms that are highlighted in red and arise due to the rigid coupling between the PCs and the BM, these forces constitute the RL-PC pathway. Since the forces $F^e_{dc/bm}$ and $F^v_{dc/bm}$ are non-zero due to the kinematic coupling terms that are highlighted in magenta and arise due to the rigid coupling between the bottom of the DCs and the BM, these forces constitute the DC pathway.

V. MODEL PARAMETERS

The values of electrical and mechanical parameters of the model that have been updated from their value in our previous paper[4] are listed in Tables S2 and S3. Parameters not listed in these tables have the same values as in Ref.[4].

TABLE S2. MET saturating conductance $G_{hb}^{max}(x)$, expressed in nS per HB. The value at other locations x are interpolated from these tabulated values.

x (cm)	Value in Ref.[4]	M1 and baseline	Adjusted value in M2	Adjusted value in M3	Adjusted value in M4	M4 with modified $G_{hb}^{max}(x)$
0	97.6	206.4	351.5	184.1	334.8	334.8
0.13	94.3	199.4	339.6	177.9	323.4	323.4
0.224	74.9	158.8	270.4	141.6	257.5	257.5
0.32	56.2	118.8	202.3	106.0	192.6	192.6
0.44	38.4	81.2	138.3	72.5	131.8	131.8
0.67	18.8	24.7	24.7	24.7	24.7	64.5
1.0						16
1.12	1.97	2.59	2.59	2.59	2.59	2.59

TABLE S3. Other updated mechanical and electrical parameters. x is the longitudinal position expressed in cm. The model includes 3 OHCs and 3 DCs per cross-section and 1000 cross-sections per cm.

Param.	Description	Value in Ref.[4]	Current value	Note
P_s^0	MET channel resting probability	0.4	0.3	Decreased to 0.3 to increase size of tonic displacement
K_{tmb} (N/m^2)	TMB stiffness (per unit length)	$3.84\exp(-7.54x)\times 10^4$	$15.4\exp(-7.54x)\times 10^4$	Was increased to improved predictions of tonic displacement at TM
K_{ohc} (N/m^2)	OHC stiffness (per unit length)	$5.07\exp(-7.54x)\times 10^3$	$20.3\exp(-7.54x)\times 10^3$	Was increased to improve predictions of tonic displacement at TM
r_{dc}	normalized DC stiffness	∞	Depends on model	
r_{rl}	normalized RL/PC joint stiffness	0.1370	Depends on model	
C_{dc} $(N.s/m^2)$	DC viscosity per unit length	0	$\frac{0.025\exp(-7.54x)}{BF(x)/BF_0}$ where $BF(x) = BF_0\exp(-4.5x)$ is the approximate expression for the best frequency of the model	Spatial variation so that phase of DC mechanical impedance ($Z_{dc} = K_{dc}/(i\omega)+C_{dc}$) evaluated at BF is approximately the same at all locations
C_{ohc} $(N.s/m^2)$	OHC viscosity per unit length	0	$= C_{dc}(x)$	assumed
M_{dc} (kg/m)	DC mass per unit length	0	$0.001\times M_{tmb}(x)$	M_{dc} was included to facilitate code implementation but was set at a low value which is effectively 0

VI. RESULTS AT MORE APICAL LOCATIONS

The results of the manuscript focus on a basal location tuned to 20 kHz ($x \approx 0.3$ cm). We show in Fig. S2 the equivalent of Fig. 3 at a more apical location tuned to 5 kHz ($x \approx 0.6$ cm depending on the model. Figure S3 shows equivalent of Fig. 4 at a more apical location tuned to 8 kHz.

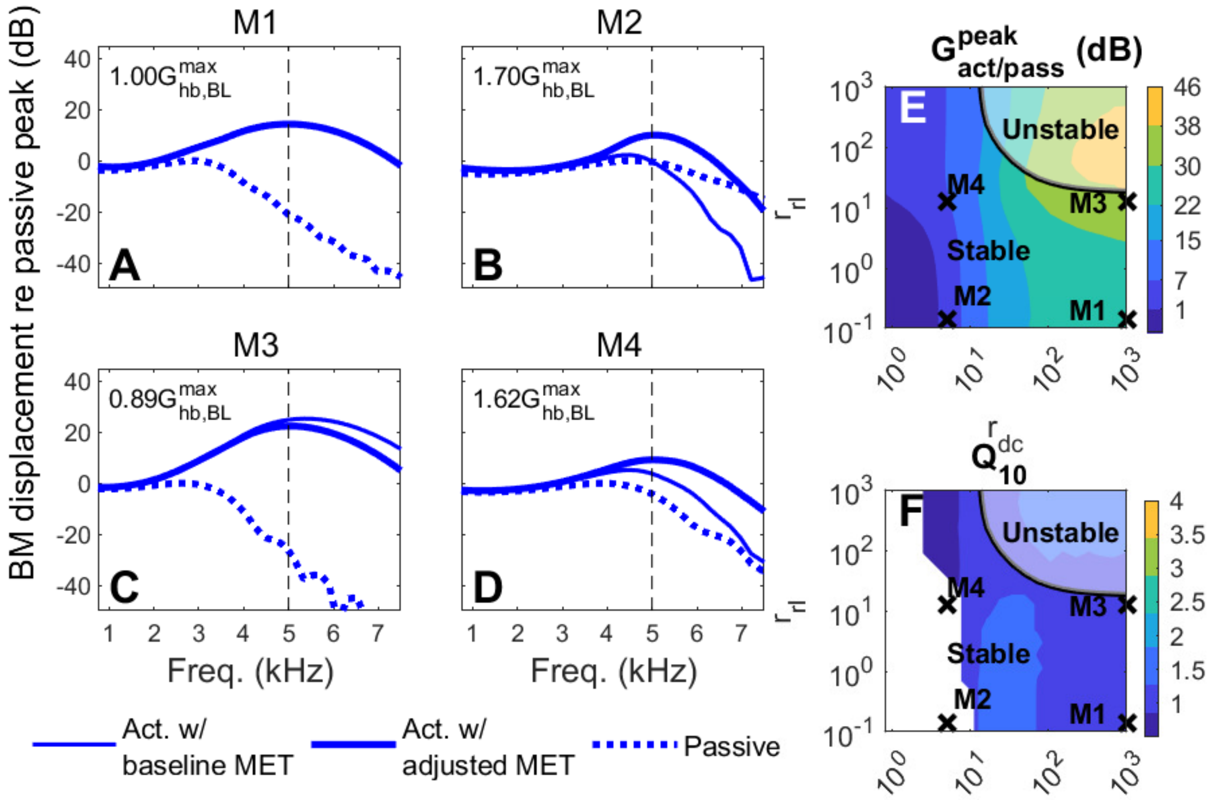

FIG. S2. Same as Fig. 3, except that the results are plotted at the location tuned to 5 kHz (x =0.66 cm in A; x =0.655 cm in B; x =0.5575 cm in C; x = 0.5725 cm in D. E and F are plotted at x= 0.66 cm. Note that the model with very low r_{DC} values are not sufficiently tuned so that a Q_{10} value cannot be calculated.

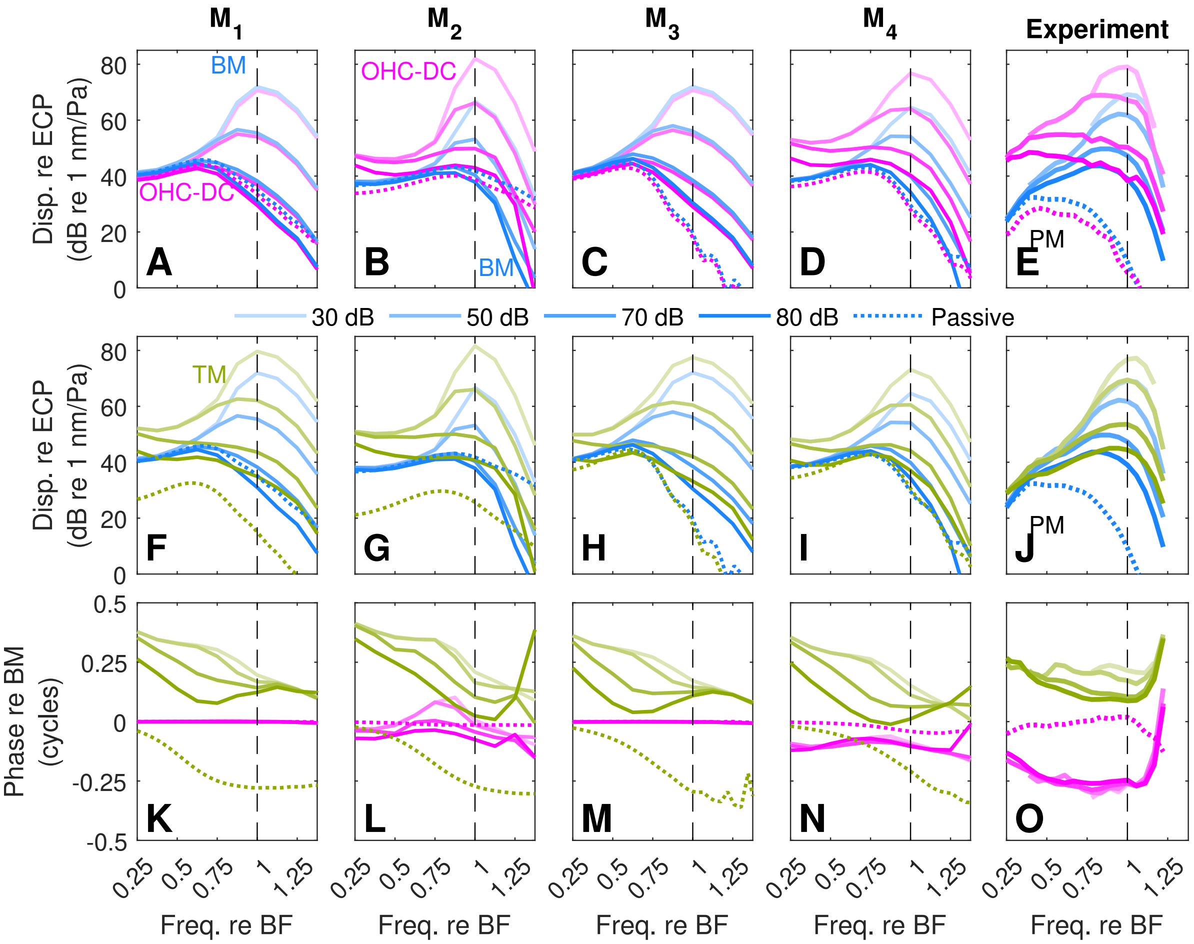

FIG. S3. Same as Fig. 4, except that the model results are plotted at the location tuned to 8 kHz (x =0.525 cm for M1; x =0.4625 cm for M2; x =0.535 cm for M3; x = 0.4925 cm for M4.)

REFERENCES

[1]Julien Meaud and Karl Grosh. The effect of tectorial membrane and basilar membrane longitudinal coupling in cochlear mechanics. *The Journal of the Acoustical Society of America*, 127(3):1411–1421, 2010.

[2]Sripriya Ramamoorthy, Niranjan V Deo, and Karl Grosh. A mechano-electro-acoustical model for the cochlea: response to acoustic stimuli. *The Journal of the Acoustical Society of America*, 121(5):2758–2773, 2007.

[3]Julien Meaud and Karl Grosh. Response to a pure tone in a nonlinear mechanical-electrical-acoustical model of the cochlea. *Biophysical journal*, 102(6):1237–1246, 2012.

[4]George Samaras, Haiqi Wen, and Julien Meaud. Broad nonlinearity in reticular lamina vibrations requires compliant organ of corti structures. *Biophysical Journal*, 122(5):880–891, 2023.
